# Implicit and Explicit Gender-Related Cognition, Gender Dysphoria, Autistic-Like Traits, and Mentalizing: Differences Between Autistic and Non-Autistic Cisgender and Transgender Adults

**DOI:** 10.1007/s10508-022-02386-5

**Published:** 2022-08-16

**Authors:** Aimilia Kallitsounaki, David M. Williams

**Affiliations:** Division of Human & Social Sciences, School of Psychology, Keynes College, University of Kent, Canterbury, CT2 7NP UK

**Keywords:** Gender cognition, Autism spectrum disorder, Transgender, Mentalizing, Autistic-like traits

## Abstract

Evidence indicates a link between autism spectrum disorder (ASD) and gender diversity, yet this intersection remains insufficiently understood. Here, we investigated whether (1) ASD affects gender-related cognition (i.e., mental processes of perceiving and interpreting one’s own gender self-concept), (2) autistic people have increased gender dysphoria and recall limited gender-typed behavior from childhood, and (3) transgender individuals have increased ASD-like traits and difficulties in mentalizing. A total of 106 non-autistic cisgender (51 birth-assigned female), 107 autistic cisgender (57 birth-assigned female), 78 non-autistic transgender (41 birth-assigned female), and 56 autistic transgender adults (27 birth-assigned female) participated in the study. The mean age of participants was 31.01 years (range = 18 to 70). Using an explicit as well as an implicit measure, for the first time, we found that ASD affected gender-related cognition only in autistic cisgender people. Sex differences were also observed in this group. Whereas autistic cisgender birth-assigned males showed a stronger implicit gender-group identification than non-autistic cisgender birth-assigned males, autistic cisgender birth-assigned females showed a weaker gender-group identification than non-autistic cisgender birth-assigned females. Furthermore, autistic cisgender people reported significantly more gender dysphoric feelings and recalled significantly less gender-typed behavior from childhood than non-autistic cisgender individuals. No difference was observed between non-autistic and autistic transgender people. We also found that relative to non-autistic cisgender individuals, both non-autistic transgender and autistic transgender people reported significantly more ASD-like traits. However, mentalizing difficulties were observed only in the latter group. This research enhances our understanding of the link between ASD and gender diversity.

**Supplementary Information:**

The online version contains supplementary material available at 10.1007/s10508-022-02386-5.

## Introduction

In recent years, clinicians and researchers have provided increasing evidence for a link between autism spectrum disorder (ASD) and gender diversity (e.g., Strang et al., 2018a; for a review and meta-analysis, see Kallitsounaki & Williams, 2022). ASD is a neurodevelopmental condition, diagnosed on the basis of behavioral difficulties with social-communication and restricted, repetitive pattern of interests and behavior (American Psychiatric Association, 2013). Gender diversity is an umbrella term used to describe a wide range of aspects of one’s experienced gender that do not correspond to one’s birth-assigned sex (Corbett et al., 2022). Among others, the term gender diversity includes people who identify with a gender other than their birth-assigned sex (i.e., transgender; Butler, 2020), including those who feel significant levels of distress about a mismatch between their experienced gender and their birth-assigned sex (i.e., gender dysphoria; American Psychiatric Association, 2013).

Research has provided strong evidence that the co-occurrence of ASD and gender diversity is accompanied by an increase in internalizing conditions (e.g., anxiety and depression), self-harm, and suicidality (George & Stokes, 2018a; Mahfouda et al., 2019; Strang et al., 2021; Strauss et al., 2021). This highlights the need for specialized and tailored support and care for this group. To achieve this, it is important to gain a holistic understanding of the intersection between ASD and gender diversity. In the current article instead of focusing on a single component of the link between ASD and gender diversity, we examined three core aspects of it in the same sample, for the first time.

### Research Question 1: Does Autism Spectrum Disorder Affect Implicit and Explicit Gender-Related Cognition?

Little is known about the mental processes involved in the formation and consolidation of gender self-concept (or gender identity) in ASD. The sparse research conducted on this topic has shown that autistic adults show a reduced propensity to explicitly identify with personality traits and gender roles that are stereotypically attributed to males (Bejerot & Eriksson, 2014; Stauder et al., 2011). It has also been found that autistic people feel less positively and identify less strongly with the gender group that matches their experienced gender than do non-autistic individuals (Cooper et al., 2018). Yet, a note of caution should be added here, as these findings are from self-report measures only. Self-report measures rely on people’s ability to represent and report their own feelings, emotions, traits, and thoughts. Given that aspects of self-awareness are considered to be divergent in ASD (e.g., Carruthers, 2009; Gopnik, 1993; Williams, 2010), it is unclear whether these findings reflect a different implicit self-experience of gender (and hence, accurate self-reporting) or difficulties in the explicit representation and/or reporting of this concept. To address this issue, along with explicit measures of gender self-concept, researchers could also use implicit measures (Wood & Eagly, 2009).

To our knowledge, no study has used an implicit measure to investigate gender-related cognition in autistic people. Adopting an individual differences approach, however, Kallitsounaki and Williams (2020a) examined how the strength of gender self-concept varies according to the number of ASD-like traits manifested by people from the general population, using not only an explicit measure, but also an Implicit Association Test (IAT; Greenwald & Farnham, 2000). It is well-established that ASD-like traits are continuously distributed in the general population and qualitatively similar to the core diagnostic characteristics of ASD (e.g., Constantino & Todd, 2003, 2005; Ronald et al., 2006; though see Mottron, 2021 for an alternative perspective).

Following this approach, Kallitsounaki and Williams (2020a) found that people from the general population with high ASD-like traits had a weaker inclination to ascribe to themselves explicitly, or identify implicitly with, feminine and masculine gender stereotypical personality traits than people with low ASD-like traits. An exploratory analysis, however, showed a selective influence of ASD-like traits on the implicit identification with gender stereotypical traits among birth-assigned females only.

The first aim of this study was to replicate these findings conceptually and extend them further. Specifically, in this study we examined gender-group identification using an explicit self-report measure and an IAT (Greenwald et al., 2002). Gender-group identification implicates people’s sense of belonging to one gender group as opposed to another and is considered as one of the two traditions of research on gender self-concept (Wood & Eagly, 2015). Based on Kallitsounaki and Williams’ findings, we made the following hypothesis:

#### *Hypothesis 1a*

The number of self-reported ASD-like traits of non-autistic cisgender people would be negatively and significantly associated with the strength of gender self-concept (more ASD-like traits = weaker explicit and implicit gender-group identification).

Next, we attempted to extend the original findings further by examining gender-group identification in autistic people. Autistic people’s sense of belonging to one gender group over another was examined in cisgender and transgender people, separately, for the first time. This enabled us to understand how exactly ASD might affect gender-related cognition. Olson et al. (2015) were the first researchers to use an IAT to examine gender-related cognition in the transgender population. They found that transgender children implicitly perceived themselves in keeping with their experienced gender. To our knowledge, no study has employed an IAT in transgender adults. On the basis of previous findings (e.g., Cooper et al., 2018; Kallitsounaki & Williams, 2020a; Olson et al., 2015), we made the following hypotheses:

#### *Hypothesis 1b*

Within each group (autistic cisgender/autistic transgender/non-autistic cisgender/non-autistic transgender) scores on the explicit and the implicit measures of gender self-concept would align with experienced gender, rather than birth-assigned sex (i.e., individuals who identify as females would show an explicit and implicit female self-concept, whereas people who identify as males would show an explicit and implicit male self-concept).

#### *Hypothesis 1c*

We expected autistic participants who identify either as cisgender or transgender to show a significantly weaker explicit and implicit gender self-concept than non-autistic people.

### Research Question 2: Do Autistic People Have Increased Gender Dysphoric Feelings and Recall Limited Gender-Typed Behavior from Childhood?

The second aim of this study was to examine whether autistic people have increased gender dysphoric feelings and whether they recall limited gender-typed behavior from their childhood years. Recent evidence indicates a small association between ASD traits and gender variance in children from the general population (Munoz Murakami et al., 2022), as well as an increased rate of gender diversity among autistic children (Corbett et al., 2022). Likewise, research among adults has shown that autistic people report a more diverse range of gender identities than non-autistic individuals (Bejerot & Eriksson, 2014; George & Stokes, 2018b) and that they are more likely to be planning or have transitioned (Cooper et al., 2018). To date, only George and Stokes (2018b) have employed a validated measure to examine gender dysphoric feelings in this population. Using the Gender Identity/Gender Dysphoria Questionnaire (GIDYQ; Deogracias et al., 2007), George and Stokes found that autistic people reported significantly more gender dysphoric feelings than non-autistic individuals. In George and Stokes’ study, however, the percentage of participants who did not identify as cisgender was 3 times higher in the autistic group than in the non-autistic group. The inclusion of these participants in the analysis could have artificially inflated the score of the autism group creating a significant difference in the number of gender dysphoric feelings between autistic and non-autistic people. To begin to answer whether autistic cisgender adults have increased gender dysphoric feelings, we examined gender dysphoric feelings among autistic cisgender and autistic transgender people, separately. We also examined, for the first time, whether these groups recall limited gender-typed behavior from childhood. On the basis of previous findings (e.g., Bejerot & Eriksson, 2014; Hisle-Gorman et al., 2019; Pecora et al., 2020; Pohl et al., 2014; Walsh et al., 2018), we made the following hypotheses:

#### *Hypothesis 2a*

We expected autistic people who identify either as cisgender or transgender to report significantly more gender dysphoric feelings than non-autistic cisgender people, autistic cisgender people to report significantly fewer gender dysphoric feelings than non-autistic transgender individuals, and autistic transgender people to report significantly more gender dysphoric feelings than non-autistic transgender individuals (autistic transgender > non-autistic transgender > autistic cisgender > non-autistic cisgender).

#### *Hypothesis 2b*

We expected autistic people who identify either as cisgender or transgender to recall significantly less gender-typed behavior from childhood than non-autistic cisgender people, autistic cisgender people to recall significantly more gender-typed behavior from childhood than non-autistic transgender individuals, and autistic transgender people to recall significantly less gender-typed behavior from childhood than non-autistic transgender individuals (autistic transgender < non-autistic transgender < autistic cisgender < non-autistic cisgender).

### Research Question 3: Do Transgender People Have Increased Autistic-Like Traits and Mentalizing Difficulties?

The third aim of the current study was to examine whether transgender people (autistic and non-autistic) have increased ASD-like traits and difficulties in mentalizing. Previous studies have suggested there may be a selective difference in ASD-like traits between transgender and control birth-assigned females only (i.e., Jones et al., 2012; Kung, 2020; Murphy et al., 2020; Vermatt et al., 2018). Similar results were also found by Nobili et al. (2018). We should note, however, that in this study cisgender participants’ score on the Autism-Spectrum Quotient (AQ-28) was higher than the score of people from the general population (Hoekstra et al., 2011). This makes the interpretation of Nobili et al.’s (2018) findings difficult. Lastly, in Pasterski et al.’s (2014) study transgender birth-assigned females, diagnosed with gender dysphoria/gender identity disorder, scored higher on the AQ-50 than non-autistic birth-assigned females, but the difference was small and nonsignificant. In contrast to previous findings, Stagg and Vincent (2019) found that transgender individuals scored significantly higher on the AQ-50 than cisgender people, regardless of participant birth-assigned sex. Likewise, using the Social Responsiveness Scale for Adults (SRS), Heylens et al. (2018) found that birth-assigned males diagnosed with gender dysphoria reported significantly more ASD-like traits than a norm group of birth-assigned males and birth-assigned females diagnosed with gender dysphoria reported significantly more ASD-like traits than a norm group of birth-assigned females. Lastly, Warrier et al. (2020) examined ASD-like traits in transgender and gender-diverse individuals using three independent samples. In all three, transgender and gender-diverse individuals reported significantly more ASD traits than cisgender people, yet sex differences were not examined.

We should note here that researchers, with the exception of Jones et al. (2012), Murphy et al. (2020), and Warrier et al. (2020), either did not control for (or did not collect/report information about) the presence of autistic people in the gender diverse samples in their studies. This is important because research has shown a high prevalence of ASD diagnoses in this population (e.g., Strauss et al., 2021; for a meta-analysis see Kallitsounaki & Williams, 2022), and as expected based on their diagnosis, autistic transgender people show significantly more ASD traits than neurotypical transgender people (Warrier et al., 2020). Arguably, the inclusion of autistic people in these studies could have inflated the AQ/SRS score of gender diverse samples. Therefore, the extent to which non-autistic transgender people have increased ASD-like traits needs further examination. Furthermore, it is still unclear whether the behavioral features of ASD in transgender people are accompanied by the cognitive difficulties that are frequently encountered in ASD and which arguably underpin the behavioral features of the condition.

ASD is characterized by well-established difficulties with mentalizing (e.g., Baron-Cohen et al., 2001a; Senju et al., 2009). Mentalizing (or Theory of Mind) is the ability to impute mental states to others (and self) in order to interpret and predict behavior (Premack & Woodruff, 1978). Mentalizing has been proposed as one of the mechanisms that could explain the link between ASD and gender diversity (Glidden et al., 2016; Jacobs et al., 2014; van der Miesen et al., 2016, 2018). To our knowledge, however, only two studies have examined mentalizing in gender diverse individuals. Stagg and Vincent (2019) did not find a significant difference in Reading the Mind in the Eyes (RMIE; Baron-Cohen et al., 2001a) performance between cisgender and transgender people. In contrast, Kung (2020) found that transgender individuals performed significantly less well on the RMIE than control participants. We should note, however, that strong conclusions cannot be drawn from Stagg and Vincent’s (2019) study because their sample was underpowered to detect a small (*d* = 0.39), but potentially meaningful, difference between transgender and cisgender people.

Another important point we should mention is that neither in Kung’s (2020) nor Stagg and Vincent’s (2019) study did researchers control for a possible/likely overrepresentation of ASD diagnoses in the transgender group. So, based on previous studies, we cannot conclude with confidence whether non-autistic transgender people show atypical mentalizing ability. This leaves a critical gap in the literature that we aimed to fill by examining ASD-like traits and mentalizing in non-autistic and autistic transgender individuals, separately. On the basis of previous findings (Walsh et al., 2018; Warrier et al., 2020), we made the following hypotheses:

#### *Hypothesis 3a*

We expected non-autistic transgender participants to report significantly more ASD-like traits than non-autistic cisgender people, but significantly fewer than autistic cisgender individuals, and autistic transgender people to report significantly more ASD-like traits than either non-autistic transgender or autistic cisgender people (autistic transgender > autistic cisgender > non-autistic transgender > non-autistic cisgender).

#### *Hypothesis 3b*

We expected non-autistic transgender individuals to perform significantly less well on the mentalizing task than non-autistic cisgender people, but significantly better than autistic cisgender people, and autistic transgender people to perform significantly less well on the task than autistic cisgender participants (autistic transgender < autistic cisgender < non-autistic transgender < non-autistic cisgender).

## Method

### Participants

A total of 106 non-autistic cisgender adults (51 birth-assigned female), 107 autistic cisgender adults (57 birth-assigned female), 78 non-autistic transgender adults (41 birth-assigned female), and 56 autistic transgender adults (27 birth-assigned female) took part in the current study. The mean age of participants was 31.01 years (range = 18 to 70). Participants who identified as gender nonconforming or unknown were excluded from the study (*n* = 4), whereas participants who identified as trans(masculine/male/female) nonbinary or masculine nonbinary (*n* = 4) were included in the transgender group. The number of birth-assigned females and males did not differ significantly between groups, χ^2^(3, *N* = 347) = 0.81, *p* = 0.846, φ = 0.05, but differences in age were found, *F*(3, 343) = 26.99, *p* < 0.001, ${\eta }_{p}^{2}$ = 0.19 (when participant groups were matched for age, results of the analyses presented below did not change substantively; see the online supplementary material). Ninety-nine percent of participants reported being native English speakers. All participants in the autism groups reported having a formal diagnosis of ASD, whereas all participants in the non-autism groups reported that they did not have a formal diagnosis of ASD. Participants were recruited via the online crowdsourcing platform Prolific Academic, social media platforms, and a database of autistic individuals interested in taking part in psychological research. All participants completed the study online after they had given written, informed consent and received compensation for their time. This study was approved by the Kent Psychology Research Ethics Committee.

The current study was preregistered on Open Science Framework (preregistration can be viewed here: https://osf.io/bke5j/?view_only=795bddddc1144ef295db601982f6a12f). We should note, however, that none of the hypotheses about the autistic transgender group have been included in the preregistration. Yet, they were all made before any statistical analyses were conducted, and if preregistered, they would be exactly the same as the ones presented in the current article (all deviations from the preregistration, as well as preregistered hypotheses and analyses that have not been included in the current manuscript are reported in the online supplementary material).

### Measures and Procedure

#### Measures of Gender Self-Concept

##### Implicit Association Test

We assessed participants’ implicit gender self-concept using the Implicit Association Test (IAT) described by Greenwald et al. (2002). Participants were instructed to sort words that belonged to one of four categories using one of two possible response keys. Categories and stimuli used in the task were (a) Self: I, me, my, mine, self; (b) Other: they, them, their, it, other; (c) Female: woman, girl, daughter, madam, lady, female; and (d) Male: man, boy, son, sir, gentleman, male.

In the first block (20 trials), the “self” category label was presented in the upper left corner of the screen and the “other” category label in the upper right corner. Participants were asked to sort words that belonged either to “self” or “other” category by pressing the “a” key of a keyboard for words related to “self” and the “l” key for words related to “other”. In the second block (20 trials), categories referred to “female” and “male” categorization. The “female” category label was presented in the upper left corner and the “male” category in the upper right corner. Participants were instructed to press the “a” key for words belonging to “female” category and the “l” key for items belonging to “male” category. In the third block (20 practice trials), the four categories were presented combined (“self/female” labels: upper left corner; “other/male” labels: upper right corner) and participants practiced the sorting task, by pressing the key that was assigned to each category in the preceding two blocks (i.e., “a” key for items belonging to “self /female” categories and “l” key for items belonging to “other/male” categories). In the fourth block (40 experimental trials), participants completed the first experimental condition of the combined sorting task. In the fifth block (40 trials), the “female” category label was presented in the upper right corner and the “male” category label in the upper left corner, subsequently the assignment of the key for each category was reversed, compared to the first block. Participants were instructed to press “l” for items belonging to the “female” category and “a” for items belonging to the “male” category. In the sixth block (20 practice trials), participants practiced the combined task using the switched key assignments. They were instructed to press the “a” key to categorize items that belonged either to the “self” or to the “male” category and the “l” key for words that belonged either to the “other” or “female” category. In the last block (40 experimental trials), they completed the second experimental condition of the combined sorting task.

The dependent measure for the IAT was the strength of the automatic associations, calculated with the scoring algorithm recommended by Greenwald et al. (2003). That is, a standardized mean difference score (namely *D*) in response latencies between the two practical blocks (i.e., blocks 3 & 6) and the two experimental blocks (i.e., bocks 4 & 7). When participants respond faster in the “self-female and other-male” condition than in the “self-male and other-female” condition, they receive a positive *D* score indicating a female self-concept. When they respond faster in the “self-male and other-female” than in the “self-female and other-male” condition they receive a negative *D* score indicating a male self-concept. When error rate in the critical blocks (i.e., practical and experimental) exceeded 20%, participants (i.e., non-autistic cisgender: *n* = 7; non-autistic transgender: *n* = 3; autistic cisgender: *n* = 14; autistic transgender: *n* = 8) were not included in the analyses (Greenwald et al., 1998; van Well et al., 2008). Results of the analyses including all participants, regardless of their performance on the task, are reported in the online supplementary material. The IAT was programmed using Inquisit Millisecond software package 4 (2015, https://www.millisecond.com), and it was administered using Inquisit Web Player 4.0.10. After the completion of the IAT, participants were automatically redirected to a Qualtrics survey to complete the rest of the tasks and questionnaires.

##### Explicit Measure

We assessed participants’ explicit gender self-concept using the measure designed by Greenwald et al. (2002). Participants were asked to rate each of the six male and six female nouns used in the IAT using a 7-point Likert scale ranging from “not at all characteristic of you” to “extremely characteristic of you.” The measure was scored by subtracting participants’ average score on the male nouns from that on the female nouns. Positive scores denote a female self-concept, and negative scores denote a male self-concept.

##### Other Tasks and Self-Report Measures

Participants were asked to indicate their birth-assigned sex, such as on an original birth certificate (i.e., male or female) and their gender identity (i.e., male, female, trans male, trans female, gender nonconforming, or other). Participants whose birth-assigned sex was congruent to their gender identity were categorized as cisgender, and those whose birth-assigned sex was incongruent to their gender identity or identified as transgender were categorized as transgender. Participants completed the RMIE (Baron‐Cohen et al., 2001a), which is a widely used measure of adult mentalizing, the AQ-50 (Baron-Cohen et al., 2001b) that measures ASD-like traits (scores ≥ 26 denote clinically significant levels of ASD-like traits), the GIDYQ (Deogracias et al., 2007), which is a reliable self-report measure that taps gender identity and gender dysphoria (scores ≤ 3 denote clinically significant levels of gender dysphoria), and the 18 items of the Recalled Childhood Gender Identity/Gender Role Questionnaire (RCGI; Zucker et al., 2006) that assess gender role behavior and gender identity. Please note that a detailed description of the tasks and measures used in this study is provided in the online supplementary material.

## Results

### Gender-Related Cognition

#### Associations Between Autism-Spectrum Quotient and Performance on the Explicit and Implicit Measures of Gender Self-Concept

In keeping with the preregistration, a series of correlation analyses was conducted to investigate the relations between AQ and performance on the explicit and implicit measures of gender self-concept among non-autistic cisgender individuals (note: scores from the explicit and implicit measures of gender self-concept were transformed to positive values, so that higher scores denote a stronger gender self-concept, regardless of whether it is male or female). As predicted, AQ score was negatively and significantly related to the strength of the explicit gender self-concept (Hypothesis 1a), *r*(104) = − 0.32, *p* < 0.001 (one-tailed). In keeping with the preregistered hypotheses, AQ score was also significantly associated with the strength of the explicit gender self-concept (note: scores from the explicit measure were untransformed) among both non-autistic cisgender birth-assigned males (more ASD-like traits = weaker male self-concept) and non-autistic cisgender birth-assigned females (more ASD-like traits = weaker female self-concept). The results of the analyses are included in the online supplementary material. Contrary to predictions, AQ did not correlate significantly with the strength of the implicit gender self-concept among non-autistic cisgender people (Hypothesis 1a), *r*(97) = − 0.06, *p* = 0.276 (one-tailed). Results remained nonsignificant when the analysis was performed for each birth-assigned sex separately (see the online supplementary material).

#### Performance on the Explicit Measure of Gender Self-Concept

A 2 (birth-assigned sex: male/female) $\times$ 2 (diagnostic category: non-autistic/autistic) $\times$ 2 (gender identity: cisgender/transgender) ANOVA was conducted on participant scores from the explicit measure of gender self-concept. Nonsignificant main effects were detected for birth-assigned sex, *F*(1, 339) = 2.18, *p* = 0.140, ${\upeta }_{p}^{2}$ = 0.01, gender identity, *F*(1, 339) = 0.05, *p* = 0.829, ${\upeta }_{p}^{2}$ = 0.00, and diagnostic category, *F*(1, 339) = 0.14, *p* = 0.707, ${\upeta }_{p}^{2}$ = 0.00. Nonetheless, the analysis revealed a significant Birth-Assigned Sex $\times$ Gender Identity interaction, *F*(1, 339) = 2843.37, *p* < 0.001, ${\upeta }_{p}^{2}$ = 0.89, a significant Birth-Assigned Sex $\times$ Diagnostic Category interaction, *F*(1, 339) = 34.46, *p* < 0.001, ${\upeta }_{p}^{2}$ = 0.09, a significant Gender Identity $\times$ Diagnostic Category interaction, *F*(1, 339) = 6.49, *p* = 0.011, ${\upeta }_{p}^{2}$ = 0.02, and a significant three-way interaction, *F*(1, 339) = 21.33, *p* < 0.001, ${\upeta }_{p}^{2}$ = 0.06.

**Fig. 1:**
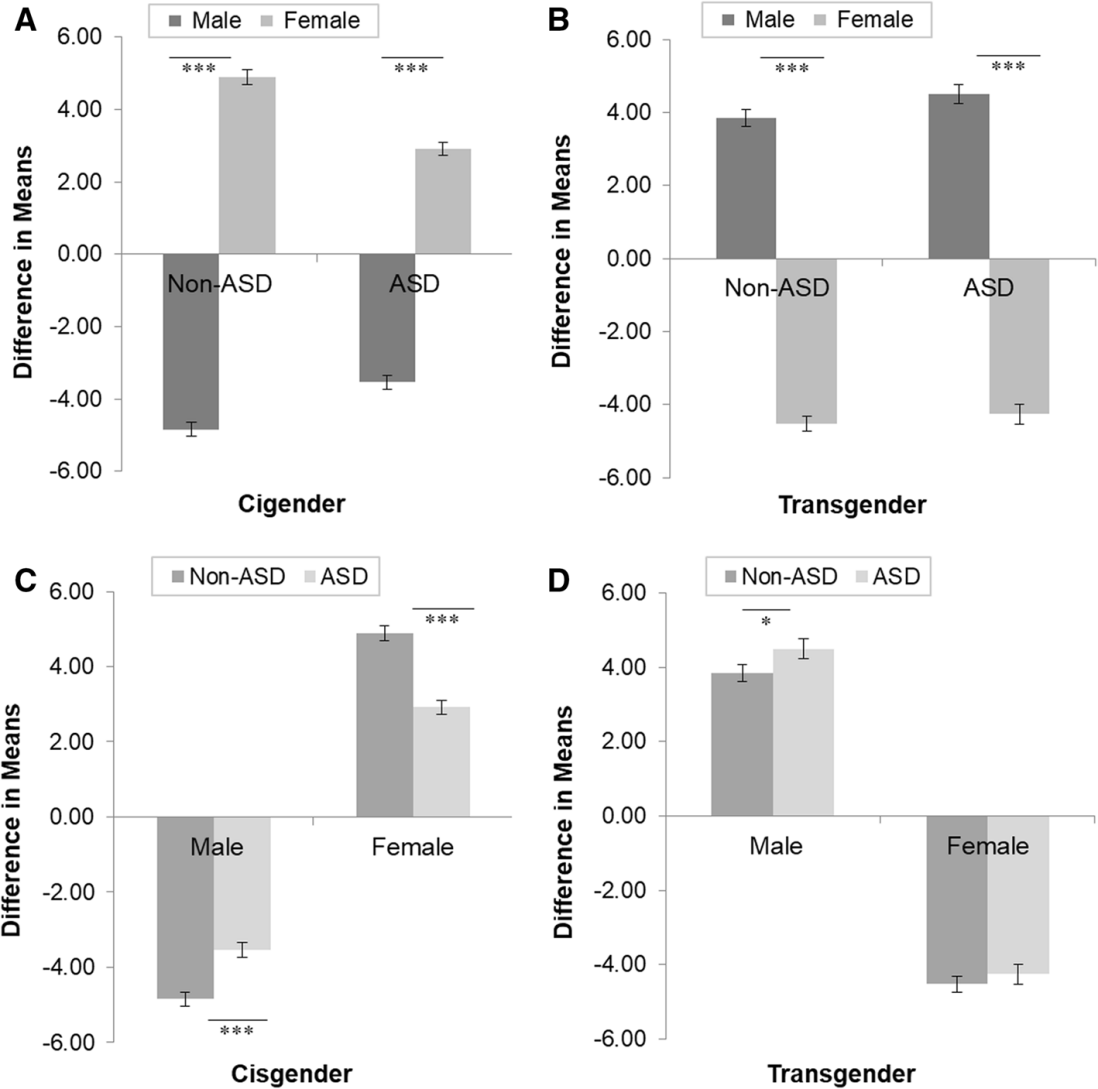
Performance on the explicit measure of gender self-concept as a function of birth-assigned sex and diagnostic category within cisgender and transgender participants *Note.* Birth-assigned males: Non-ASD cisgender *n* = 55; ASD cisgender *n* = 50; Non-ASD transgender *n* = 37; ASD transgender *n* = 29. Birth-assigned females: Non-ASD cisgender *n* = 51; ASD cisgender *n* = 57; Non-ASD transgender *n* = 41; ASD transgender *n* = 27. For *p*s < 0.001, ${\eta }_{p}^{2}$ ≥ 0.06; for *p* < 0.05, ${\eta }_{p}^{2}$ = 0.01; for *p* > 0.05, ${\eta }_{p}^{2}$ = 0.00. *p < 0.05 (one-tailed). *** *p* < 0.001 (one-tailed)

Breaking down the three-way interaction, a simple effects analysis of birth-assigned sex within gender identity and diagnostic category indicated that in accordance with the preregistered Hypothesis 1b, the explicit measure of gender self-concept was sensitive to gender identity differences. Results of the analysis are illustrated in Fig. 1A and B. Specifically, we found that among non-autistic and autistic cisgender individuals, birth-assigned females scored significantly *higher* than birth-assigned males (note: mean scores of birth-assigned females were significantly above zero, and mean scores of birth-assigned males were significantly below zero, all *p*s < 0.001, one-tailed). This indicates that birth-assigned females who identify as females showed an explicit female self-concept and birth-assigned males who identify as males showed an explicit male self-concept. The opposite pattern was observed among non-autistic and autistic transgender individuals, with birth-assigned females scoring significantly lower than birth-assigned males (note: mean scores of birth-assigned females were significantly below zero, and mean scores of birth-assigned males were significantly above zero, all *p*s < 0.001, one-tailed). This indicates that birth-assigned females who identify as males showed an explicit male self-concept in line with their experienced (rather than birth-assigned) gender, whereas birth-assigned males who identify as females showed an explicit female self-concept in line with their experienced (rather than birth-assigned) gender. These results were entirely expected.

Next, we conducted a simple effects analysis of diagnostic category within birth-assigned sex and gender identity. The results of the analysis are illustrated in Fig. 1C and D. In line with the preregistered Hypothesis 1c, autistic cisgender birth-assigned males scored significantly higher on the explicit task than non-autistic cisgender birth-assigned males, and autistic cisgender birth-assigned females scored significantly lower on the task than non-autistic cisgender birth-assigned females. This indicates a weaker explicit gender self-concept among autistic cisgender participants than among non-autistic cisgender participants. Contrary to predictions, we found that autistic transgender birth-assigned males scored significantly higher on the explicit task than non-autistic transgender birth-assigned males. This shows that autistic transgender birth-assigned males showed a stronger explicit female self-concept than non-autistic transgender birth-assigned males. Also unexpectedly, no difference in the strength of the explicit gender self-concept was observed between autistic transgender birth-assigned females and non-autistic transgender birth-assigned females. Thus, autistic and non-autistic transgender birth-assigned females displayed an explicit male gender self-concept that was in keeping with their experienced gender, rather than birth-assigned gender, to the same degree.

#### Performance on the Implicit Measure of Gender Self-Concept

A 2 (birth-assigned sex: male/female) $\times$ 2 (diagnostic category: non-autistic/autistic) $\times$ 2 (gender identity: cisgender/transgender) ANOVA was conducted on participant scores from the IAT. Significant main effects were detected for birth-assigned sex, *F*(1, 304) = 6.41, *p* = 0.012, ${\upeta }_{p}^{2}$ = 0.02, gender identity, *F*(1, 304) = 7.64, *p* = 0.006, ${\upeta }_{p}^{2}$ = 0.03, and diagnostic category, *F*(1, 304) = 13.83, *p* < 0.001, ${\upeta }_{p}^{2}$ = 0.04. The analysis also yielded a significant Birth-Assigned Sex $\times$ Gender Identity interaction, *F*(1, 304) = 302.40, *p* < 0.001, ${\upeta }_{p}^{2}$ = 0.50 and a significant Gender Identity $\times$ Diagnostic Category interaction, *F*(1, 304) = 4.98, *p* = 0.026, ${\upeta }_{p}^{2}$ = 0.02. Neither the Birth-Assigned Sex $\times$ Diagnostic Category interaction, *F*(1, 304) = 0.15, *p* = 0.701, ${\upeta }_{p}^{2}$ = 0.00, nor the three-way interaction, *F*(1, 304) = 2.80, *p* = 0.095, ${\upeta }_{p}^{2}$ = 0.01 were significant.

**Fig. 2:**
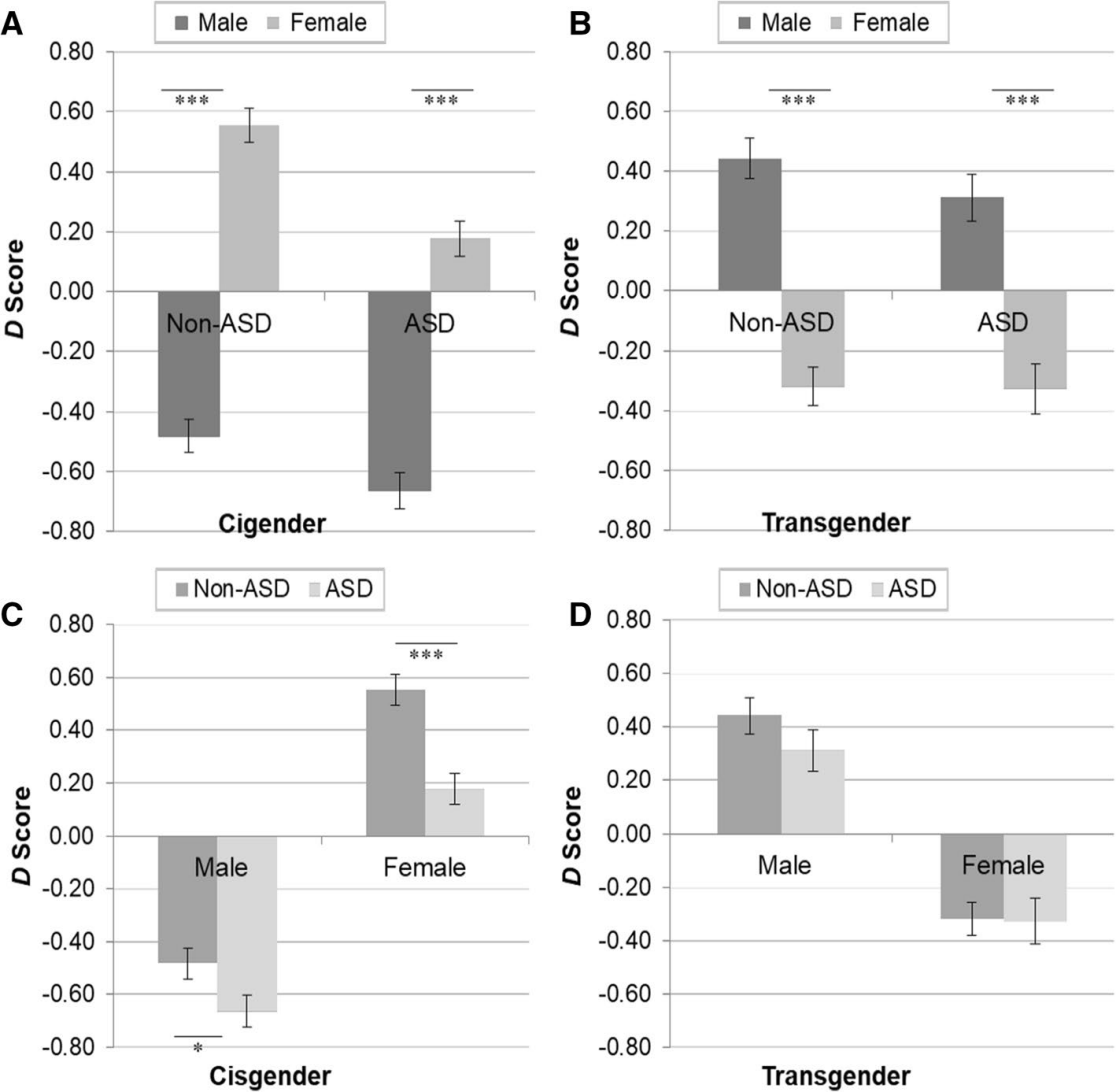
Performance on the implicit measure of gender self-concept as a function of birth-assigned sex and diagnostic category within cisgender and transgender participants *Note.* Birth-assigned males: Non-ASD cisgender *n* = 50; ASD cisgender *n* = 43; Non-ASD transgender *n* = 34; ASD transgender *n* = 26. Birth-assigned females: Non-ASD cisgender *n* = 49; ASD cisgender *n* = 48; Non-ASD transgender *n* = 40; ASD transgender *n* = 22. For *p*s < 0.001, ${\eta }_{p}^{2}$ ≥ 0.07; for *p* < 0.05, ${\eta }_{p}^{2}$ = 0.02; for *p*s > 0.05, ${\eta }_{p}^{2}$ ≤ 0.01 *p < 0.05 (one-tailed). *** *p* < 0.001 (one-tailed)

Breaking down the three-way interaction, a simple effects analysis of birth-assigned sex within gender identity and diagnostic category indicated that in accordance with the preregistered Hypothesis 1b, the IAT was sensitive to gender identity differences. Results of the analysis are illustrated in Fig. 2A and B. Specifically, we found that among non-autistic and autistic cisgender individuals, birth-assigned females scored significantly higher than birth-assigned males (note: mean scores of birth-assigned females were significantly above zero (non-autistic *p* < 0.001, one-tailed; autistic *p* = 0.003, one-tailed), and mean scores of birth-assigned males were significantly below zero (all *p*s < 0.001, one-tailed). This indicates that birth-assigned females who identify as females showed an implicit female self-concept, whereas birth-assigned males who identify as males showed an implicit male self-concept. The opposite pattern was observed among non-autistic and autistic transgender individuals, with birth-assigned females scoring significantly lower than birth-assigned males (note: mean scores of birth-assigned females were significantly below zero, and mean scores of birth-assigned males were significantly above zero, all *p*s < 0.001, one-tailed). This indicates that birth-assigned females who identify as males showed an implicit male self-concept in line with their experienced (rather than birth-assigned) gender, and birth-assigned males who identify as females showed an implicit female self-concept in line with their experienced (rather than birth-assigned) gender. These results were entirely expected.

Next, we conducted a simple effects analysis of Diagnostic Category within Birth-assigned Sex and Gender Identity. The results of the analysis are illustrated in Fig. 2C and D. In line with the preregistered Hypothesis 1c, we found that autistic cisgender birth-assigned females achieved a significantly lower *D* score on the IAT than non-autistic cisgender birth-assigned females, indicating a weaker implicit female self-concept. Contrary to predictions, we found that autistic cisgender birth-assigned males achieved a significantly lower *D* score on the IAT than non-autistic cisgender birth-assigned males, indicating a stronger implicit male self-concept. Also unexpectedly, a nonsignificant difference in the strength of implicit gender self-concept was found between autistic and non-autistic transgender individuals. Both autistic and non-autistic transgender adults displayed an implicit gender self-concept that was in keeping with their experienced gender, rather than birth-assigned gender, to the same degree.

### Performance on Reading the Mind in the Eyes and Self-Report Measures

Table 1 shows descriptive statistics for participant scores on GIDYQ, RCGI, AQ, and RMIE, and Table 2 shows the results of a series of ANOVAs we conducted on these scores.

**Table 1:** Participant characteristics and mean (SD) performance on Reading the Mind in the Eye and self-report measures

Groups	Age	RMIE	GIDYQ ^a,b^	RCGI ^a,c^	AQ
	*M* (*SD*)	*M* (*SD*)	*M* (*SD*)	*M* (*SD*)	*M* (*SD*)
Non-autistic cis	37.10 (12.89)	26.09 (4.65)	4.80 (0.20)	3.94 (0.52)	19.98 (7.42)
Non-autistic trans	26.51 (9.02)	26.59 (3.77)	2.16 (0.32)	2.63 (0.63)	24.55 (9.24)
Autistic cis	31.55 (7.86)	19.61 (7.80)	4.18 (0.71)	3.52 (0.63)	31.45 (7.21)
Autistic trans	24.73 (6.85)	24.66 (4.70)	2.23 (0.45)	2.58 (0.64)	35.11 (6.70)

Cis Cisgender, *Trans *Transgender, *Age *Measured in years, *RMIE *Reading the Mind in the Eyes (0–36), *GIDYQ *Gender Identity/Gender Dysphoria Questionnaire (1–5), *RCGI *Recalled Childhood Gender Identity/Gender Role Questionnaire (1–5), *AQ *Autism-Spectrum Quotient (0–50)

^a^One non-autistic cisgender birth-assigned male completed the female version of the GIDYQ and RCGI, and one autistic cisgender birth-assigned female completed the male version of the GIDYQ and RCGI. Hence, their data has not been included in the analysis

^b^Low scores = more gender dysphoria. ^c^ Low scores = less recalled gender-typed behavior from childhood

**Table 2:** 2 (Birth-assigned Sex: Male/Female) × 2 (Diagnostic Category: Non-ASD/ASD) × 2 (Gender Identity: Cisgender/Transgender) ANOVA results

Measure	Effect	*F*	*p*	${\eta }_{p}^{2}$	Direction of main effects
GIDYQ	Sex	12.55	< 0.001	0.04	Birth-assigned females < Birth-assigned males
	Gender identity	1947.98	< 0.001	0.85	Transgender < Cisgender
	Diagnostic category	28.73	< 0.001	0.08	Autistic < Non-autistic
	Gender identity $\times$ Diagnostic category	41.11	< 0.001	0.11	
	Sex $\times$ Gender identity	3.35	0.068	0.01	
	Sex $\times$ Diagnostic category	0.78	0.378	0.00	
	Sex $\times$ Gender identity $\times$ Diagnostic category	0.10	0.756	0.00	
RCGI	Sex	92.67	< 0.001	0.22	Birth-assigned females < Birth-assigned males
	Gender identity	361.98	< 0.001	0.52	Transgender < Cisgender
	Diagnostic category	16.63	< 0.001	0.05	Autistic < Non-autistic
	Gender identity $\times$ Diagnostic category	7.37	0.007	0.02	
	Sex $\times$ Gender identity	1.10	0.295	0.00	
	Sex $\times$ Diagnostic category	0.83	0.364	0.00	
	Sex $\times$ Gender identity $\times$ Diagnostic category	0.99	0.322	0.00	
AQ	Sex	3.32	0.069	0.01	Birth-assigned females = Birth-assigned males
	Gender identity	22.79	< 0.001	0.06	Transgender > Cisgender
	Diagnostic category	167.92	< 0.001	0.33	Autistic > Non-autistic
	Gender identity $\times$ Diagnostic category	0.21	0.645	0.00	
	Sex $\times$ Gender identity	2.67	0.103	0.01	
	Sex $\times$ Diagnostic category	0.26	0.608	0.00	
	Sex $\times$ Gender identity $\times$ Diagnostic category	0.34	0.563	0.00	
RMIE	Sex	7.50	0.006	0.02	Birth-assigned females < Birth-assigned males
Measure	Effect	*F*	*p*	${\eta }_{p}^{2}$	Direction of the main effects
	Gender identity	19.02	< 0.001	0.05	Transgender > Cisgender
	Diagnostic category	45.40	< 0.001	0.12	Autistic < Non-autistic
	Gender identity $\times$ Diagnostic category	12.63	< 0.001	0.04	
	Sex $\times$ Gender identity	2.33	0.128	0.01	
	Sex $\times$ Diagnostic category	7.34	0.007	0.02	
	Sex $\times$ Gender identity $\times$ Diagnostic category	1.59	0.209	0.01	

*Sex *Sex-assigned at birth, *GIDYQ *Gender Identity/Gender Dysphoria Questionnaire, *RCGI *Recalled Childhood Gender Identity/Gender Role Questionnaire, *AQ *Autism-Spectrum Quotient, *RMIE *Reading the Mind in the Eyes

#### Gender Dysphoric Feelings

A 2 (birth-assigned sex: male/female) $\times$ 2 (diagnostic category: non-autistic/autistic) $\times$ 2 (gender identity: cisgender/transgender) ANOVA was conducted on GIDYQ scores. Significant main effects were detected for birth-assigned sex, such that birth-assigned females (marginal *M* = 3.25, *SE* = 0.04) reported significantly more gender dysphoric feelings than birth-assigned males (marginal *M* = 3.44, *SE* = 0.04), gender identity, such that transgender individuals (marginal *M* = 2.20, *SE* = 0.04) reported significantly more gender dysphoric feelings than cisgender people (marginal *M* = 4.49, *SE* = 0.03), and diagnostic category, such that autistic people (marginal *M* = 3.21, *SE* = 0.04) reported significantly more gender dysphoric feelings than non-autistic individuals (marginal *M* = 3.48, *SE* = 0.04). The analysis also revealed a significant Gender Identity $\times$ Diagnostic Category interaction. The Birth-Assigned Sex $\times$ Gender Identity interaction was nonsignificant, as were the Birth-Assigned Sex $\times$ Diagnostic Category interaction and the three-way interaction.

To test Hypothesis 2a, the significant Gender Identity $\times$ Diagnostic Category interaction was treated as a 4-level variable and a series of planned *t*-tests was conducted. Results of the analyses are presented in Table 3. As predicted, autistic cisgender people reported significantly more gender dysphoric feelings than non-autistic cisgender people, but significantly fewer than non-autistic and autistic transgender participants. Contrary to predictions, there was no significant difference in GIDYQ score between non-autistic and autistic transgender individuals (hence, autistic transgender = non-autistic transgender < autistic cisgender < non-autistic cisgender).

**Table 3:** Planned comparisons between groups

Measure	*t*-tests	Cohen’s *d*	95% CI
*GIDYQ*			
	Non-autistic cis > Non-autistic trans ***	10.18	[9.09, 11.27]
	Non-autistic cis > Autistic cis ***	1.18	[0.88, 1.47]
	Non-autistic cis > Autistic trans ***	8.37	[7.39, 9.34]
	Non-autistic trans < Autistic cis ***	− 3.47	[− 3.93, − 3.01]
	Non-autistic trans = Autistic trans	− 0.18	[− 0.52, 0.17]
	Autistic cis > Autistic trans ***	3.08	[2.61, 3.54]
*RCGI*			
	Non-autistic cis > Non-autistic trans ***	2.30	[1.92, 2.67]
	Non-autistic cis > Autistic cis ***	0.73	[0.45, 1.01]
	Non-autistic cis > Autistic trans ***	2.42	[2.00, 2.83]
	Non-autistic trans < Autistic cis ***	− 1.41	[− 1.74, − 1.08]
	Non-autistic trans = Autistic trans	0.08	[− 0.26, 0.43]
	Autistic cis > Autistic trans ***	1.49	[1.13, 1.85]
*AQ*			
	Non-autistic cis < Non-autistic trans ***	− 0.56	[− 0.85, − 0.26]
	Non-autistic cis < Autistic cis ***	− 1.57	[− 1.87, − 1.26]
	Non-autistic cis < Autistic trans ***	− 2.11	[− 2.50, − 1.71]
	Non-autistic trans < Autistic cis ***	− 0.85	[− 1.15, − 0.54]
	Non-autistic trans < Autistic trans ***	− 1.28	[− 1.65, − 0.90]
	Autistic cis < Autistic trans **	− 0.52	[− 0.85, − 0.19]
*RMIE*			
	Non-autistic cis = Non-autistic trans	− 0.12	[− 0.41, 0.18]
	Non-autistic cis > Autistic cis ***	1.01	[0.72, 1.29]
	Non-autistic cis > Autistic trans * ^a^	0.31	[− 0.02, 0.63]
	Non-autistic trans > Autistic cis ***	1.09	[0.77, 1.40]
	Non-autistic trans > Autistic trans **	0.46	[0.11, 0.81]
	Autistic cis < Autistic trans ***	− 0.73	[− 1.06, − 0.40]

*Cis* Cisgender, *Trans* Transgender, *GIDYQ* Gender Identity/Gender Dysphoria Questionnaire, *RCGI *Recalled Childhood Gender Identity/Gender Role Questionnaire, *AQ *Autism-spectrum Quotient, 95% *CI *95% Confidence Intervals

^a^Please note that when groups were matched for age the *p* value was less than 0.01 (one-tailed) and the size of the between-group difference was medium (i.e., *d* = 0.54). *p < 0.05. **p < 0.01. ***p < 0.001

#### Recalled Gender-Typed Behavior from Childhood

A 2 (birth-assigned sex: male/female) $\times$ 2 (diagnostic category: non-autistic/autistic) $\times$ 2 (gender identity: cisgender/transgender) ANOVA was conducted on RCGI scores. Significant main effects were detected for birth-assigned sex, such that birth-assigned females (marginal *M* = 2.89, *SE* = 0.04) recalled significantly less gender-typed behavior from childhood than birth-assigned males (marginal *M* = 3.46, *SE* = 0.04), gender identity, such that transgender participants (marginal *M* = 2.61, *SE* = 0.05) recalled significantly less gender-typed behavior from childhood than cisgender participants (marginal *M* = 3.73, *SE* = 0.04), and diagnostic category, such that autistic participants (marginal *M* = 3.05, *SE* = 0.04) recalled significantly less gender-typed behavior than non-autistic participants (marginal *M* = 3.29, *SE* = 0.04). The analysis also revealed a significant Gender Identity $\times$ Diagnostic Category interaction. The Birth-Assigned Sex $\times$ Gender Identity interaction was nonsignificant, as were the Birth-Assigned Sex $\times$ Diagnostic Category interaction and the three-way interaction.

To test Hypothesis 2b, the significant Gender Identity $\times$ Diagnostic Category interaction was treated as a 4-level variable and a series of planned *t*-tests was conducted. Results of the analyses are presented in Table 3. As predicted, autistic cisgender people recalled significantly less gender-typed behavior from childhood than non-autistic cisgender people, but significantly more than non-autistic and autistic transgender participants. Contrary to predictions, there was no significant difference in RCGI score between non-autistic and autistic transgender individuals (hence, autistic transgender = non-autistic transgender > autistic cisgender > non-autistic cisgender).

#### Autism-like Traits

A 2 (birth-assigned sex: male/female) $\times$ 2 (diagnostic category: non-autistic/autistic) $\times$ 2 (gender identity: cisgender/transgender) ANOVA was conducted on AQ scores. Significant main effects were detected for gender identity, such that transgender participants (marginal *M* = 29.82, *SE* = 0.67) reported significantly more ASD-like traits than cisgender participants (marginal *M* = 25.74, *SE* = 0.53), and diagnostic category, such that autistic participants (marginal *M* = 33.32, *SE* = 0.63) reported significantly more ASD-like traits than non-autistic participants (marginal *M* = 22.24, *SE* = 0.57). The main effect of birth-assigned sex was nonsignificant, as were the Birth-Assigned Sex $\times$ Gender Identity interaction, the Birth-Assigned Sex $\times$ Diagnostic Category interaction, the Gender Identity $\times$ Diagnostic Category interaction, and the three-way interaction.

To examine Hypothesis 3a, a series of planned *t*-tests was conducted. Results of the analyses are presented in Table 3. As predicted, non-autistic transgender participants reported significantly more ASD-like traits than non-autistic cisgender people, but significantly fewer than autistic cisgender individuals. We also found that autistic transgender individuals reported significantly more ASD-like traits than autistic cisgender people (hence, autistic transgender > autistic cisgender > non-autistic transgender > non-autistic cisgender).

#### Mentalizing Ability

A 2 (birth-assigned sex: male/female) $\times$ 2 (diagnostic category: non-autistic/autistic) $\times$ 2 (gender identity: cisgender/transgender) ANOVA was conducted on RMIE scores. Significant main effects were detected for birth-assigned Sex, such that birth-assigned males (marginal *M* = 25.11, *SE* = 0.44) performed significantly better on the task than birth-assigned females (marginal *M* = 23.43, *SE* = 0.43), gender identity, such that transgender participants (marginal *M* = 25.61, *SE* = 0.48) performed significantly better on the task than cisgender participants (marginal *M* = 22.93, *SE* = 0.38), and diagnostic category, such that non-autistic participants (marginal *M* = 26.34, *SE* = 0.41) performed significantly better on the task than autistic participants (marginal *M* = 22.20, *SE* = 0.46). The analysis also revealed a significant Birth-Assigned Sex $\times$ Diagnostic Category interaction and a significant Gender Identity $\times$ Diagnostic Category interaction. Neither the Birth-Assigned Sex $\times$ Gender Identity interaction nor the three-way interaction were significant.

To test Hypothesis 3b, the significant Gender Identity $\times$ Diagnostic Category interaction was treated as a 4-level variable and a series of planned *t*-tests was conducted. Results of the analyses are presented in Table 3. In contrast to predictions, no differences emerged in RMIE task performance between non-autistic transgender and non-autistic cisgender people, but as expected, both groups performed significantly better than autistic cisgender people. In keeping with predictions, autistic transgender participants scored significantly lower on the task than both non-autistic cisgender and non-autistic transgender people, but contrary to predictions, they scored significantly higher than autistic cisgender people (hence, autistic cisgender < autistic transgender < non-autistic transgender = non-autistic cisgender).

## Discussion

In the current study we examined three core aspects of the link between ASD and gender diversity as a whole, aiming to acquire a more holistic understanding of intersectional identities (autistic + gender diverse). We found that relative to non-autistic cisgender people, autistic cisgender people showed a less strong explicit identification with gender groups that corresponded to their experienced gender (and that autistic cisgender birth-assigned females, but not males, showed a less strong implicit identification, too). This was accompanied by fewer memories of gender-typed behavior in childhood and increased (albeit not clinically significant) levels of current gender dysphoric feelings. In contrast, autistic transgender people showed a strong identification with gender groups that matched their experienced gender, and their levels of childhood gender-typed behavior and gender dysphoria were equivalent to levels reported by non-autistic transgender people. Lastly, the latter group presented elevated ASD-like traits. However, these traits were not accompanied by challenges with mentalizing. Below follows a more extensive discussion of the findings.

### Implicit and Explicit Gender-Related Cognition in Autism Spectrum Disorder

The first aim of the current study was to examine whether ASD affects gender-related cognition. To do so, we first investigated the relation between ASD-like traits, on the one hand, and the strength of explicit and implicit gender self-concept, on the other hand, among non-autistic cisgender people. The approach followed was taken by Kallitsounaki and Williams (2020a) to enable our attempt to replicate (conceptually) their findings. In keeping with predictions, AQ score was negatively and significantly associated with the strength of the explicit gender self-concept. Results indicate that non-autistic cisgender people with elevated ASD-like traits have a weaker propensity to explicitly self-identify with gender groups. This finding adds to recent evidence provided by Kallitsounaki and Williams (2020a) that cisgender people with high ASD-like traits display a weaker inclination to explicitly identify with personality traits that stereotypically characterize either females or males. As levels of ASD-like traits increase, so too may be reporting the experience of a gender self-concept that it is not strongly associated with either feminine/masculine attributes or a collective gender identity. One possible explanation for this association is that people with high ASD traits might feel less obliged to conform to the societal expectations of how one’s own gender should be presented at a behavioral level. This could be attributed to a reduced experience of self-conscious emotions (e.g., pride and guilt; Davidson et al., 2017). Of course, further research is required to test this hypothesis.

Contrary to predictions, the number of self-reported ASD-like traits was not significantly associated with the strength of the implicit gender self-concept. This finding is inconsistent with that of Kallitsounaki and Williams (2020a) who observed a significant association between number of ASD-like traits and the strength of the implicit gender self-concept. Thus, contrary to the results of the current study, Kallitsounaki and Williams found the higher the ASD-like traits among cisgender people the weaker the automatic identification of self with either masculine or feminine personality traits. One possible explanation for why the current study failed to replicate the results of the original study is that in this study we used an IAT that taps implicit self-identification with gender groups, whereas Kallitsounaki and Williams (2020a) used an IAT that taps implicit self-identification with gender-stereotypical personality traits. Although both approaches have been traditionally used in gender identity research, they are not equivalent, as both have derived from different theoretical and research backgrounds (Wood & Eagly, 2015). This should be taken into account in future gender-related studies that use an IAT. It could be the case that non-autistic individuals with high ASD-like traits implicitly identify with gender-stereotypical traits to a lesser degree than non-autistic individuals with low ASD-like traits, but nonetheless, show no difference in implicit identification with gender groups.

Next, we aimed to extend Kallitsounaki and Williams’ (2020a) findings further by investigating, for the first time, gender-related cognition in autistic cisgender and transgender people, using an explicit and implicit measure of gender self-concept. As expected, both measures tapped the experienced gender of participants. Specifically, among cisgender individuals, either non-autistic or autistic, birth-assigned females showed an explicit and implicit female self-concept and birth-assigned males showed an explicit and implicit male self-concept. Whereas, among transgender individuals, either non-autistic or autistic, birth-assigned females who identify as males showed an explicit and implicit male self-concept and birth-assigned males who identify as females showed an explicit and implicit female self-concept.

To our knowledge, this is the first study to investigate implicit gender self-concept in transgender adults and found that non-autistic and autistic transgender adults perceive themselves explicitly and implicitly in terms of their experienced gender. Olson et al. (2015) rightly, in our view, highlighted that “the IAT should not be seen as a lie detector test” (p. 468). Taken together, however, results from the explicit and implicit task, it can be argued that the current study might provide counter evidence to the hypothesis that symptoms of gender dysphoria in ASD (e.g., cross-dressing) reflect more an obsession that arises from autistic people’s inherent predisposition toward unusual interests and preoccupations than a “genuine” mismatch between their experienced gender and their birth-assigned sex (Parkinson, 2014; Tateno et al., 2008). Furthermore, results indicate that autistic people (cisgender and transgender) are able to formulate a gender self-concept, regardless of whether it matches their birth-assigned sex. It is unclear, however, whether autistic and non-autistic people identify with their experienced gender to the same degree. To answer to this question, we first compared the strength of explicit and implicit gender self-concept between autistic and non-autistic cisgender people.

As predicted, autistic cisgender individuals (birth-assigned males and females) showed a weaker explicit identification with the gender groups associated with their birth-assigned sex than non-autistic cisgender people. This was expected, given previous findings of lower explicit gender identification in autistic people (Cooper et al., 2018) and people with increased ASD-like traits (Kallitsounaki & Williams, 2020a). Furthermore, on the basis of previous findings within the general population (Kallitsounaki & Williams, 2020a), we expected autistic cisgender females to show a significantly lower score on the implicit measure of gender self-concept than non-autistic cisgender females. Indeed, our hypothesis was confirmed. This important finding is the first of its kind, to our knowledge, and suggests that autistic cisgender females have a weaker inclination to incorporate into their self-concept a collective gender concept that matches their birth-assigned sex. We also predicted that autistic cisgender males would show a significantly lower score on the implicit measure of gender self-concept than non-autistic cisgender males. Unexpectedly, however, autistic cisgender males displayed a significantly higher score on the implicit measure of gender self-concept than non-autistic cisgender males, indicating a stronger inclination to incorporate into their self-concept a collective gender concept that matches their birth-assigned sex. Thus, there is an important effect of sex here.

Autistic cisgender females show weaker implicit and explicit identification with female gender groups than non-autistic cisgender females. In contrast, autistic cisgender males show weaker explicit, yet stronger implicit identification with male gender groups than non-autistic cisgender males. The observed mismatch between their explicit and implicit experience of male self-concept among autistic cisgender males might reflect a difference in self-awareness. The idea that there are differences in self-awareness between autistic and non-autistic people has been expressed by many (e.g., Frith & Happé, 1999; Hobson, 1990; Williams, 2010), but to our knowledge, sex differences in self-awareness have not been examined in the autistic population and results from studies in the general population remain inconclusive (e.g., Jonsson & Allwood, 2003; Lemieux et al., 2019; Prentice & Murphy, 2022).

Next, we compared the strength of explicit and implicit gender self-concept between autistic transgender and non-autistic transgender individuals. Contrary to predictions, autistic transgender and non-autistic transgender birth-assigned females identified explicitly with the gender groups associated with their experienced gender to the same degree, and autistic transgender birth-assigned males identified explicitly with the gender groups of their experienced gender more strongly than non-autistic transgender birth-assigned males. Also unexpectedly, the performance of autistic transgender individuals (birth-assigned males and females) on the implicit measure of gender self-concept did not differ significantly from the performance of non-autistic transgender individuals. Both groups identified implicitly with their experienced gender, rather than their birth-assigned gender, to the same degree.

In sum, autistic cisgender people were able to identify explicitly with the gender groups of their birth-assigned sex, yet they identified less strongly than did non-autistic cisgender people. They also identified implicitly with the gender groups associated with their birth-assigned sex, but the strength of the identification was weaker only among birth-assigned females. In contrast, autistic transgender and non-autistic transgender people identified with their experienced gender at least to the same degree, both explicitly and implicitly. On this basis, it could be argued that these results provide preliminary evidence of a selective influence of ASD in the consolidation of a collective gender self-concept that matches people’s birth-assigned sex.

To put it simply, ASD seems to hinder the explicit (and implicit among birth-assigned females) identification of people only with the gender groups associated with their birth-assigned sex. The explicit and implicit identification of autistic transgender people with the gender groups of their experienced gender seems to be unaffected by ASD. It is possible that the formation and consolidation of a gender self-concept that does not correspond to birth-assigned sex might be achieved through trajectories and mechanisms that are different from the ones followed when a gender self-concept corresponds to birth-assigned sex. To get a better understanding of the findings from the autistic adult population (cisgender and transgender), we believe it is essential to know how gender self-concepts develops in autistic children. Given that research on this topic is almost nonexistent, future research will be needed to elucidate whether the development of gender self-concept follows the same cognitive and developmental trajectories in autistic and non-autistic children (van Schalkwyk et al., 2015). The second overarching aim of this study was to investigate the extent to which the strength of gender-group identification autistic people show is in line with their current gender dysphoric feelings and recalled childhood gender-typed behavior.

### Gender Dysphoric Feelings and Recalled Gender-Typed Behavior in Autism Spectrum Disorder

We first examined whether autistic transgender people report increased current gender dysphoric feelings and recall limited gender-typed behavior from childhood. To our knowledge, this is the first study that attempted to answer this question. We found that in accordance to the mismatch autistic transgender people expressed between their birth-assigned sex and their experienced gender, they reported clinically significant levels of gender dysphoria and they recalled limited gender-typed behavior from childhood. This indicates that autistic transgender and non-autistic transgender people feel an extreme distress of their body, anatomy, and function to the same degree and that feelings related to gender diversity had an early onset in both groups. Taken together, these findings provide further support to the clinical recommendation that adequate support and care should be provided for transgender people regardless of whether they have a co-occurring diagnosis of ASD (e.g., Strang et al., 2018b).

Next, we examined whether autistic cisgender people report increased current gender dysphoric feelings and recall diminished gender-typed behavior from childhood. In keeping with George and Stokes’ (2018b) findings, we found that autistic cisgender individuals reported significantly more gender dysphoric feelings than non-autistic cisgender people, but significantly less than autistic and non-autistic transgender people. We also extended this finding further by showing, for the first time, that autistic cisgender people recalled less gender-typed behavior in their childhood memories than non-autistic cisgender individuals, but more than autistic and non-autistic transgender people. This is in line with recent findings that autistic people report a more diverse range of gender identities and are more likely to be gender diverse and to have or be planning a gender transition than non-autistic people (Bejerot & Eriksson, 2014; Cooper et al., 2018; George & Stokes, 2018b). This also supports the hypothesis that there is link between ASD and gender diversity (e.g., Strang et al., 2018a). Nonetheless, we should stress that the mechanisms that could underpin this link are still unclear and further research is required. Given the risks involved in this type of research such endeavors might be best supported by community involvement (e.g., community-based participatory approaches to help direct and contextualize such research). Epidemiological studies are also required to get an estimate of the size of this link. To date, the only study of the prevalence of gender dysphoria diagnosis in the autistic population has been conducted among children (Hisle-Gorman et al., 2019). The hypothesis of a link between ASD and gender diversity has also been supported by evidence of increased ASD traits in gender diverse people (Kallitsounaki & Williams, 2022). Therefore, the last overarching aim of this study was to examine whether transgender individuals have increased ASD-like traits and/or difficulties in mentalizing ability.

### Autistic-like Traits and Mentalizing Ability in Transgender Individuals

As predicted, non-autistic transgender individuals reported significantly more ASD-like traits than non-autistic cisgender individuals, but significantly fewer than autistic people (either cisgender or transgender). This is important because to date, only a very small number of studies (i.e., Jones et al., 2012; Murphy et al., 2020; Warrier et al., 2020) have examined whether increased ASD-like traits are observed in samples of non-autistic gender diverse adults. In this study we also found that autistic transgender people reported significantly more ASD-like traits than autistic cisgender individuals. This is in keeping with previous research findings (Walsh et al., 2018; Warrier et al., 2020), but further research is required to understand this finding. If high scores on the AQ tap solely ASD characteristics in both autistic cisgender and autistic transgender groups, then these results indicate an increased severity of ASD in autistic transgender people. If this is true, autistic transgender people should also score higher on standardized diagnostic tools for ASD (e.g., Autism Diagnostic Observation Schedule; Lord et al., 2000) than autistic cisgender people. To our knowledge, this has not been examined in the literature.

We should highlight here that it remains debatable whether ASD-like traits observed among gender diverse people tap “true” ASD characteristics (Fortunato et al., 2022; Turban, 2018; Turban & van Schalkwyk, 2018). It has been suggested that the psychological impact of stigma, marginalization, and rejection that transgender people frequently experience increase their liability to develop features that “mimic” ASD characteristics (Fortunato et al., 2022; Turban, 2018; Turban & van Schalkwyk, 2018). To investigate this hypothesis we examined whether the behavioral features of ASD observed among transgender individuals (non-autistic and autistic) were accompanied by (traditionally-observed) difficulties at the cognitive level. If ASD-like traits among non-autistic and autistic transgender people reflected true ASD characteristics, then these groups should show the mentalizing difficulty that is frequently observed in ASD.

Interestingly, this was not the case. Non-autistic transgender people’s performance on the mentalizing task was equivalent to the performance of non-autistic cisgender individuals. This is out of keeping with Kung (2020) who found that transgender males had significantly poorer mentalizing ability than cisgender females and transgender females poorer mentalizing ability than cisgender males. Nonetheless, as discussed above, Kung (2020) did not control for the presence of autistic participants in the transgender sample. Furthermore, we found, for the first time, that autistic transgender people scored significantly lower on the RMIE than non-autistic cisgender and non-autistic transgender people, indicating a mentalizing difficulty. Noteworthy, non-autistic transgender people performed significantly better on the task than autistic cisgender people.

From a theoretical perspective, these results add to the discussion as to whether ASD-like traits in transgender people represent true ASD characteristics (Fortunato et al., 2022; Turban, 2018; Turban & van Schalkwyk, 2018). We found that non-autistic transgender people showed increased ASD-like traits, albeit intact mentalizing abilities. In contrast, autistic transgender people showed not only increased ASD-like traits, but also potential mentalizing difficulties. Based on these results, it could be argued that ASD-like traits in non-autistic transgender people do not reflect “true” ASD characteristics, whereas they do tap ASD among autistic transgender individuals. Furthermore, research has shown a significant relation between mentalizing ability and gender dysphoric feelings among people from the general population (Kallitsounaki & Williams, 2020b; Kallitsounaki et al., 2021). Yet, the findings of the current study indicate that this relation does not hold among non-autistic transgender people and thus contradict the hypothesis that mentalizing is the shared underling mechanism that explains the high co-occurrence of ASD and gender diversity (Glidden et al., 2016; Jacobs et al., 2014; van der Miesen et al., 2016). Based on previous findings, however, we cannot exclude the possibility that mentalizing might play a role in the development of subclinical gender dysphoric feelings in autistic cisgender people (Kallitsounaki & Williams, 2020b; Kallitsounaki et al., 2021). Of course, further research and replication of the current findings is required before major conclusions can be made. To increase confidence in the veracity of the current findings, future research might usefully examine mentalizing abilities in transgender (autistic and non-autistic) children as well as in their parents and siblings, employing not only explicit, but also implicit measures of mentalizing (e.g., Senju et al., 2009).

### Limitations and Directions for Future Research

Although the findings of this study enhance our understanding of the high co-occurrence between ASD and gender diversity by elucidating some core aspects of this phenomenon, we should note potential limitations in this study. In order for autistic participants to take part in the current study, they had to report possession of a formal diagnosis of ASD. We cannot exclude the possibility, however, that self-diagnosed individuals also took part in the study. Future online studies should account for the issue of sample identifiability (i.e., internally valid, but not necessarily representative, results; Rubenstein & Furnie, 2021), by asking participants to provide a copy of their diagnostic letter (see Warrier et al., 2020). Despite this potential limitation, it is important to mention that autistic participants in this study showed clinical levels of ASD, as well as mentalizing difficulties. The presentation of ASD diagnosis is the autistic group of the current study was in keeping with that found in other studies where not only evidence of a formal diagnosis of ASD has been provided by autistic participants, but also an independent validation of their diagnosis has been made (e.g., Nicholson et al., 2019).

Furthermore, in this study we used two dimensional measures, one that measures current gender dysphoric feelings (i.e., GIDYQ) and one that taps recalled gender-typed behavior in childhood (i.e., RCGI). Although both measures are commonly used in research, they do not capture non-binary gender identification that is common in ASD (e.g., Walsh et al., 2018). Future research might usefully employ measures (e.g., Genderqueer Identity scale; McGuire et al., 2019) that tap gender identification beyond binary categories.

We should also acknowledge that in the current study we did not collect information on co-occurring mental health conditions. Given that co-occurring mental health conditions are prevalent in both autistic and transgender populations (e.g., Dhejne et al., 2016; Lai et al., 2019), and might have an impact on some of the findings of the current study (e.g., increased ASD traits in non-autistic transgender individuals), future research might address this limitation.

Lastly, we should note that some of the stimuli in the “Other” category of the IAT were in fact gender neutral pronouns (e.g., they, them, etc.). Given that gender neutral pronouns are commonly used by gender diverse people, it could be argued that this methodological limitation might have attenuated the scores of transgender participants on the IAT. We should note, however, that in the current study we included mostly participants who identified with a binary gender, and thus they were more likely to use female or male pronouns than neutral pronouns. Also the IAT task is designed to be easier when two strongly associated concepts (e.g., Self and Experienced Gender) share the same response option than when two unrelated or only weakly related concepts share the same response option (e.g., Self and Other Gender; Nosek et al., 2007). On this basis, we would expect even a transgender male who uses gender neutral pronouns to respond faster and more accurately when words related to self (e.g., I, me, mine) share the same response key with gender groups that correspond to their experienced gender (e.g., male, son, sir, etc.) than when words related to self share the same response key with gender groups of the opposite binary gender (e.g., girl, female, madam, etc.). Of course, in future studies it might be useful to collect information on pronoun use when employing IATs that tap self-concepts. This would provide a clear answer on whether the use of neutral pronouns might have an effect on participant score.

### Conclusion

In sum, the main findings of this study indicate that **(a)** ASD appears to hinder the consolidation of a strong explicit (and implicit among birth-assigned females) gender self-concept in autistic cisgender people only, **(b)** autistic cisgender people have increased current gender dysphoric feelings and recall limited gender-typed behavior from childhood, and **(c)** non-autistic transgender people show only the behavioral features of ASD, whereas autistic transgender people show features of ASD at the behavioral and cognitive level. The current study enhances our understanding of the link between ASD and gender diversity. We hope future research will further extend the evidence base required to better inform clinical judgement and improve best practice.

## Supplementary Information

Below is the link to the electronic supplementary material.

Supplementary file1 (DOCX 85 KB)

## Data Availability

This study was preregistered on Open Science Framework (https://osf.io/bke5j). The data that support the findings of this study are available from the corresponding author, Aimilia Kallitsounaki, upon reasonable request.

## Code availability

N/A
