# Supplementary material for: Implicit and Explicit Gender-Related Cognition, Gender Dysphoria, Autistic-Like Traits, and Mentalizing: Differences Between Autistic and Non-Autistic Cisgender and Transgender Adults
Source: Arch Sex Behav. 2022 Aug 16;51(7):3583–600. doi: 10.1007/s10508-022-02386-5 (PMC9556420; doi:10.1007/s10508-022-02386-5)
Supplement: Supplementary file 1 — Supplementary file1 (DOCX 85 KB) [file 10508_2022_2386_MOESM1_ESM.docx]

**Supplementary Material**

1. **Matching Participant Groups for Age and Statistical Analyses**

To verify that the between-group differences on the key dependent variables reported in the manuscript resulted from differences in diagnostic status (autistic/non-autistic) and gender identity (transgender/cisgender), rather than from between-group differences in age, we matched participant groups closely on this variable. Following the recommendation of Mervis and Klein-Tasman (2004), groups were considered matched only if between-group differences in age were not statistically significant (i.e., *p* ≥ .500). In order to achieve this matching, we gradually removed the oldest non-autistic cisgender participants and the youngest autistic cisgender participants. Then, we gradually removed the oldest non-autistic transgender participants and the youngest autistic transgender participants, and finally we gradually removed the oldest non-autistic and autistic cisgender participants and the youngest non-autistic and autistic transgender participants, until groups were well-matched for age (see Table S1). The number of birth-assigned females and males did not differ significantly between groups, χ^2^ (3, *N* = 219) = 5.92, *p* = .115, φ = .16. As shown below, results remained essentially the same when groups were matched for age.

| **Table S1** | | | | | | | | |
| --- | --- | --- | --- | --- | --- | --- | --- | --- |
| *Descriptive and Matching Statistics* | | | | | | | | |
| Variable | Groups | | | | Comparisons | | |  |
|  | Non-autistic cis  *n* = 68 | Autistic cis  *n* = 69 | Non-autistic trans  *n* = 52 | Autistic trans  *n* = 30 | *t*-tests | *p* | Cohen’s *d* | 95% CI |
|  | *M*(*SD*) | *M*(*SD*) | *M*(*SD*) | *M*(*SD*) |  |  |  |  |
| Age | 28.84  (5.83) | 29.16 (2.84) | 28.50  (7.32) | 28.93  (6.94) |  |  |  |  |
|  |  |  |  |  | Non-autistic cis = Non-autistic trans | .779 | 0.05 | [-0.31, 0.41] |
|  |  |  |  |  | Non-autistic cis = Autistic cis | .683 | -0.07 | [-0.41, 0.27] |
|  |  |  |  |  | Non-autistic cis = Autistic trans | .944 | -0.02 | [-0.45, 0.41] |
|  |  |  |  |  | Non-autistic trans = Autistic cis | .540 | -0.13 | [-0.49, 0.24] |
|  |  |  |  |  | Non-autistic trans = Autistic trans | .793 | -0.06 | [-0.51, 0.39] |
|  |  |  |  |  | Autistic cis = Autistic trans | .864 | 0.05 | [-0.38, 0.48] |
| *Note.* Cis = cisgender; Trans = transgender; 95% CI = 95% Confidence Intervals. | | | | | | | | |

***Statistical Analyses***

**Performance on the Explicit Measure of Gender Self-Concept.**

A 2 (birth-assigned sex: male/female) $\times$ 2 (diagnostic category: non-autistic/autistic) $\times$ 2 (gender identity: cisgender/transgender) ANOVA was conducted on participant scores from the explicit measure of gender self-concept. The three-way interaction remained significant, *F*(1, 211) = 10.51, *p* = .001, $\eta_{p}^{2}$ = .05, and the results of the simple effects analysis did not change substantively (i.e., from significant to nonsignificant and vice versa). Just as in the unmatched sample, the simple effects analysis of birth-assigned sex within gender identity and diagnostic category indicated that the explicit measure of gender self-concept was sensitive to gender identity differences (all *p*s < .001, one-tailed; all$\eta_{p}^{2}$ ≥ .59).

Next, a simple effects analysis of diagnostic category within birth-assigned sex and gender identity was conducted. Results of the analyses did not change substantively. Just as in the unmatched sample, autistic cisgender birth-assigned males (marginal *M* = -3.78, *SE* = 0.27) scored significantly higher on the task than non-autistic cisgender birth-assigned males (marginal *M* = -4.82, *SE* = 0.22), *F*(1, 211) = 8.79, *p* = .001 (one-tailed), $\eta_{p}^{2}$ = .04, and autistic cisgender birth-assigned females (marginal *M* = 2.80, *SE* = 0.22) scored significantly lower than non-autistic cisgender birth-assigned females (marginal *M* = 5.01, *SE* = 0.26), *F*(1, 211) = 42.97, *p* < .001 (one-tailed), $\eta_{p}^{2}$ = .17. Among transgender participants, autistic birth-assigned males (marginal *M* = 4.87, *SE* = 0.36) scored significantly higher on the explicit task than non-autistic birth-assigned males (marginal *M* = 3.85, *SE* = 0.23), *F*(1, 211) = 5.30, *p* = .011 (one-tailed), $\eta_{p}^{2}$ = .03, and no difference in the strength of the explicit gender self-concept was observed between autistic (marginal *M* = -3.99, *SE* = 0.36) and non-autistic birth-assigned females (marginal *M* = -4.37, *SE* = 0.30), *F*(1, 211) = 0.67, *p* = .207 (one-tailed), $\eta_{p}^{2}$ = .00.

**Performance on the Implicit Measure of Gender Self-Concept.**

A 2 (birth-assigned sex: male/female) $\times$ 2 (diagnostic category: non-autistic/autistic) $\times$ 2 (gender identity: cisgender/transgender) ANOVA was conducted on participant scores from the IAT. The three-way interaction remained nonsignificant, *F*(1, 184) = 0.99, *p* = .322, $\eta_{p}^{2}$ = .01, and the results of the simple effects analysis did not change substantively. Just as in the unmatched sample, the simple effects analysis of birth-assigned sex within gender identity and diagnostic category indicated that the IAT was sensitive to gender identity differences (all *p*s < .001, one-tailed; all$\eta_{p}^{2}$ ≥ .08).

Next, a simple effects analysis of diagnostic category within birth-assigned sex and gender identity was conducted. Just as in the unmatched sample, autistic cisgender females (marginal *M* = 0.18, *SE* = 0.07) achieved a significantly lower *D* score on the IAT than non-autistic cisgender females (marginal *M* = 0.44, *SE* = 0.08), *F*(1,184) = 6.64, *p* = .006 (one-tailed), $\eta_{p}^{2}$ = .04, and autistic cisgender males (marginal *M* = -0.76, *SE* = 0.09) achieved a significantly lower *D* score than non-autistic cisgender males (marginal *M* = -0.48, *SE* = 0.07), *F*(1, 184) = 6.87, *p* = .005 (one-tailed), $\eta_{p}^{2}$ = .04. Furthermore, the size of the *D* score did not differ significantly either between autistic (marginal *M* = -0.33, *SE* = 0.12) and non-autistic transgender birth-assigned females (marginal *M* = -0.47, *SE* = 0.09), *F*(1, 184) = 0.89, *p* = .173 (one-tailed), $\eta_{p}^{2}$ = .01, or between autistic (marginal *M* = 0.30, *SE* = 0.12) and non-autistic transgender birth-assigned males (marginal *M* = 0.44, *SE* = 0.08), *F*(1, 184) = 0.93, *p* = .168 (one-tailed), $\eta_{p}^{2}$ = .01.

**Performance on RMIE and Self-Report Measures.**

A series of 2 (birth-assigned sex: male/female) $\times$ 2 (diagnostic category: non-autistic/autistic) $\times$ 2 (gender identity: cisgender/transgender) ANOVAs was conducted on RMIE, AQ, GIDYQ, and RCGI scores. Table S2 shows descriptive statistics for participant scores. Just as in the unmatched sample, none of the 3-way interactions were significant (all *p*s ≥ .349, all $\eta_{p}^{2}$ ≤ .005) and as shown in Table S3, results of the *t*-tests did not change substantively.

| **Table S2**  *Participant Characteristics and Mean (Standard Deviation) Performance on RMIE and Self-Report Measures* | | | | |
| --- | --- | --- | --- | --- |
| Groups | RMIE | GIDYQ ^a,b^ | RCGI ^a,c^ | AQ |
|  | *M* (*SD*) | *M* (*SD*) | *M* (*SD*) | *M* (*SD*) |
| Non-autistic cis | 26.29 (4.61) | 4.78 (0.18) | 3.93 (0.48) | 20.75 (7.64) |
| Non-autistic trans | 27.02 (4.23) | 2.17 (0.33) | 2.71 (0.61) | 24.96 (9.29) |
| Autistic cis | 18.25 (8.20) | 4.12 (0.76) | 3.50 (0.59) | 30.61 (6.76) |
| Autistic trans | 23.73 (5.18) | 2.25 (0.49) | 2.53 (0.66) | 37.50 (6.21) |
| *Note.* Cis = cisgender; Trans = transgender; RMIE = Reading the Mind in the Eyes; GIDYQ = Gender Identity/Gender Dysphoria Questionnaire; RCGI =Recalled Childhood Gender Identity/Gender Role Questionnaire; AQ = Autism-Spectrum Quotient.  ^a^ One non-autistic cisgender male completed the female version of the GIDYQ and RCGI, and one autistic cisgender female completed the male version of the GIDYQ and RCGI. Hence, their data has not been included in the analysis. ^b^ Low scores = more gender dysphoria. ^c^ Low scores = less recalled gender-typed behavior from childhood. | | | | |

| **Table S3** | | | |
| --- | --- | --- | --- |
| *Planned and Post-Hoc Comparisons Between Groups* | | | |
| Measure | *t*-tests | Cohen’s *d* | 95% CI |
| GIDYQ |  |  |  |
|  | Non-autistic cis > Non-autistic trans *** | 10.06 | [8.72, 11.39] |
|  | Non-autistic cis > Autistic cis *** | 1.20 | [0.83, 1.56] |
|  | Non-autistic cis > Autistic trans *** | 8.22 | [6.97, 9.45] |
|  | Non-autistic trans < Autistic cis *** | -3.19 | [-3.73, -2.64] |
|  | Non-autistic trans = Autistic trans | -0.20 | [-0.65, 0.26] |
|  | Autistic cis > Autistic trans *** | 2.73 | [2.14, 3.30] |
| RCGI |  |  |  |
|  | Non-autistic cis > Non-autistic trans *** | 2.27 | [1.81, 2.73] |
|  | Non-autistic cis > Autistic cis *** | 0.81 | [0.46, 1.16] |
|  | Non-autistic cis > Autistic trans *** | 2.59 | [2.03, 3.15] |
|  | Non-autistic trans < Autistic cis *** | -1.32 | [-1.72, -0.92] |
|  | Non-autistic trans = Autistic trans | 0.29 | [-0.16, 0.74] |
|  | Autistic cis > Autistic trans *** | 1.58 | [1.10, 2.06] |
| AQ |  |  |  |
|  | Non-autistic cis < Non-autistic trans ** | -0.50 | [-0.87, -0.13] |
|  | Non-autistic cis < Autistic cis *** | -1.37 | [-1.74, -0.99] |
|  | Non-autistic cis < Autistic trans *** | -2.31 | [-2.85, -1.77] |
|  | Non-autistic trans < Autistic cis *** | -0.71 | [-1.08, -0.34] |
|  | Non-autistic trans < Autistic trans *** | -1.51 | [-2.01, -1.00] |
|  | Autistic cis < Autistic trans *** | -1.04 | [-1.49, -0.59] |
| RMIE |  |  |  |
|  | Non-autistic cis = Non-autistic trans | -0.16 | [-0.52, 0.20] |
|  | Non-autistic cis > Autistic cis *** | 1.21 | [0.84, 1.57] |
|  | Non-autistic cis > Autistic trans ** | 0.54 | [0.10, 0.97] |
|  | Non-autistic trans > Autistic cis *** | 1.29 | [0.90, 1.69] |
|  | Non-autistic trans > Autistic trans ** | 0.72 | [0.25, 1.18] |
|  | Autistic cis < Autistic trans *** | -0.74 | [-1.18, -0.30] |
| *Note.* Cis = cisgender; Trans = transgender; RMIE = Reading the Mind in the Eyes test; AQ = Autism-Spectrum Quotient; GIDYQ = Gender Identity/Gender Dysphoria Questionnaire; RCGI =Recalled Childhood Gender Identity/Gender Role Questionnaire; 95% CI = 95% Confidence Intervals.  **p* < .05. ***p* < .01. ****p* < .001. | | | |

1. **List and Explanation of Deviations from the Preregistration Document in the Full Paper**

| **Where?** | **What?** | **Why?** |
| --- | --- | --- |
| Sample size | - *N* = 347 instead of 306, mainly because of the inclusion of a fourth participant group that was not planned at preregistration. Hence, non-autistic cisgender adults *n* = 106 instead of 102, non-autistic transgender adults *n* = 78 instead of 102, autistic cisgender adults *n* = 107 instead of 102, and autistic transgender adults *n* = 56 (not included in the preregistration). | - After data collection begun, but *before* any statistical analyses were conducted, we decided to include as an independent group those autistic participants who identified as transgender and those transgender participants who reported a diagnosis of ASD, rather than exclude them from the study. - Due to changes in the data collection procedures (see below) preselection of participants based on specific criteria (diagnosis of ASD, gender identity etc.) was not always feasible. This explains partially why the total number of participants and the number of participants per group deviate from the preregistered numbers. |
| Hypotheses | - Hypotheses about autistic transgender people were made before any statistical analyses were conducted. Therefore, *p* values for one-tailed tests are reported. - Preregistration Block 5: Negative association between explicit/implicit gender self-concept, and GIDYQ and RCGI among transgender people (autistic and non-autistic instead of positive. | - Due to the inclusion of autistic transgender people in the study as an independent group. - Due to a typographical error in the preregistration. |
| Study design | - 2 (birth-assigned sex: male/female) × 2 (diagnostic category: non-ASD/ASD) × 2 (gender identity: cisgender/transgender) instead of 3 (group: non-ASD/ ASD/transgender) x 2 (sex: male/female). | - Due to the inclusion of an additional group (i.e., autistic transgender). |
| Data collection procedures | - Autistic participants were recruited through social media, Prolific Academic, and the Autism Research at Kent database. - Autistic participants (non-autistic and transgender) received £7.50 (i.e., the equivalent of £10 per hour) for their participation instead of £4. - Although, one of the inclusion criteria was that participants must have English as their first language, three nonnative English speakers took part in the study | - The planned collaboration with City, University of London did not proceed. - The completion time of the study was increased to 45 minutes to meet the needs of autistic people. - Preselection of participants based on their language was not always feasible during the data collection. |
| Data exclusion | - Participants with error rate > 20% in the critical blocks of the IAT were excluded from the analysis. | - We adopted the analytic strategy followed by Greenwald et al. (1998) to exclude the possibility that poor performance on the task had an effect on the results of the study. We decided to adopt this approach *before* conducting any inferential statistical analysis. |
| Analysis | - To examine differences in mentalizing, ASD-like traits, current gender dysphoria, and recalled gender-typed behavior, a series of 2 (birth-assigned sex: male/female) × 2 (diagnostic category: non-ASD/ASD) × 2 (gender identity: cisgender/transgender) ANOVAs were conducted instead of one-ways AVOVAs. - Fisher’s Z tests described in Blocks 5 and 7 of the preregistration were not conducted. The moderation analysis described in Block 7 was also not conducted. | - Due to the inclusion of an additional group (i.e., autistic transgender) and because it has become increasingly common to examine sex/gender-specific effects in studies on ASD and gender diversity. - Relations between scores from the implicit measure of gender self-concept, and GIDYQ and RCGI were mostly out of keeping with predictions, so the results of Fisher’s Z test would not be informative. Relations between AQ, GIDYQ, and RMIE were mostly out of keeping with our predictions, so a moderation analysis could not be conducted and the results of Fisher’s Z tests would not be informative. |

1. **Preregistered Hypotheses and Analyses not Included in the Manuscript**

***Block 1.3:*** ***Implicit Gender Self-Concept***

*Hypothesis 1.* Among birth-assigned males and females, we expected non-autistic cisgender individuals to show a significantly stronger implicit gender self-concept than autistic cisgender, non-autistic transgender, and autistic transgender people. We also expected autistic cisgender people to show a significantly stronger implicit gender self-concept than both non-autistic and autistic transgender individuals. Lastly, we predicted that non-autistic transgender people would show a significantly stronger implicit gender self-concept than autistic transgender people (non-autistic cisgender > autistic cisgender > non-autistic transgender > autistic transgender).

**Analysis.**

A 2 (birth-assigned sex: male/female) $\times$ 2 (diagnostic category: non-autistic/autistic) $\times$ 2 (gender identity: cisgender/transgender) ANOVA was conducted on participant scores from the IAT (note: *D* scores were transformed to positive values, so that the higher the score the stronger the implicit gender self-concept, regardless of whether it is male or female. Also, participants with error rate > 20% in the critical blocks of the task were excluded from the analysis). As shown in Table S4, the 3-way interaction was significant. To test our hypotheses, a series of planned *t*-tests was conducted. Results of the analyses are presented in Table S4.

| **Table S4** | | | | | | | |
| --- | --- | --- | --- | --- | --- | --- | --- |
| *Implicit Measure of Gender Self-Concept Analysis of Variance Results and Planned t-Tests* | | | | | | | |
| Measure | Effect | *F* | *p* | 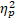 | Planned *t-*tests ^a^ | Cohen’s *d* | 95% CI |
| Implicit | Sex | 3.83 | .051 | .01 |  |  |  |
|  | Gender identity | 8.18 | .005 | .03 |  |  |  |
|  | Diagnostic category | 2.77 | .097 | .01 |  |  |  |
|  | Sex $\times$ Gender identity | 1.85 | .175 | .01 |  |  |  |
|  | Sex $\times$ Diagnostic category | 6.04 | .015 | .02 |  |  |  |
|  | Gender identity $\times$ Diagnostic category | 0.06 | .814 | .00 |  |  |  |
|  | Sex $\times$ Gender identity$\times$ Diagnostic category | 14.22 | <.001 | .05 |  |  |  |
|  |  |  |  |  | **Birth-Assigned Males** |  |  |
|  |  |  |  |  | Non-autistic cis = Non-autistic trans | 0.08 | [-0.36, 0.52] |
|  |  |  |  |  | Non-autistic cis < Autistic cis ** | -0.50 | [-0.91, -0.08] |
|  |  |  |  |  | Non-autistic cis = Autistic trans | 0.39 | [-0.09, 0.87] |
|  |  |  |  |  | Non-autistic trans < Autistic cis ** | -0.55 | [-1.01, -0.09] |
|  |  |  |  |  | Non-autistic trans = Autistic trans | 0.30 | [-0.21, 0.81] |
|  |  |  |  |  | Autistic cis > Autistic trans *** | 0.84 | [0.33, 1.35] |
|  |  |  |  |  | **Birth-Assigned Females** |  |  |
|  |  |  |  |  | Non-autistic cis > Non-autistic trans ** | 0.69 | [0.26, 1.12] |
|  |  |  |  |  | Non-autistic cis > Autistic cis *** | 1.01 | [0.58, 1.43] |
|  |  |  |  |  | Non-autistic cis > Autistic trans ** | 0.70 | [0.18, 1.22] |
|  |  |  |  |  | Non-autistic trans = Autistic cis | 0.32 | [-0.10, 0.75] |
|  |  |  |  |  | Non-autistic trans = Autistic trans | 0.01 | [-0.51, 0.54] |
|  |  |  |  |  | Autistic cis = Autistic trans | -0.31 | [-0.81, 0.20] |
| *Note.* Sex = birth-assigned sex; Cis = cisgender; Trans = transgender.  ^a^ Symbols for statistical significance denote results from one-tailed tests.  ***p* < .01. ****p* < .001. | | | | | | | |

In contrast to predictions, autistic cisgender birth-assigned males (*n* = 43; *M* = 0.68; *SD* = 0.32) achieved a significantly higher score on the IAT than non-autistic cisgender birth-assigned males (*n* = 50; *M* = 0.53; *SD* = 0.29). This indicates a stronger implicit gender self-concept among autistic cisgender birth-assigned males than among non-autistic cisgender birth-assigned males. Also, a nonsignificant difference in the strength of the implicit gender self-concept was found between non-autistic cisgender birth-assigned males and non-autistic transgender birth-assigned males (*n* = 34; *M* = 0.51; *SD* = 0.31), and between non-autistic cisgender and autistic transgender birth-assigned males (*n* = 26; *M* = 0.41; *SD* = 0.32). As predicted, however, autistic cisgender birth-assigned males scored significantly higher on the task than both non-autistic transgender and autistic transgender birth-assigned males. Results indicate a stronger implicit gender self-concept among autistic cisgender birth-assigned males than among non-autistic transgender and autistic transgender birth-assigned males. Contrary to predictions, a nonsignificant difference in the strength of the implicit gender self-concept was found between non-autistic transgender and autistic transgender birth-assigned males.

In keeping with predictions, non-autistic cisgender birth-assigned females (*n* = 49; *M* = 0.63; *SD* = 0.28) scored significantly higher on the task than non-autistic transgender (*n* = 40; *M* = 0.44; *SD* = 0.28), autistic cisgender (*n* = 48; *M* = 0.35; *SD* = 0.29), and autistic transgender birth-assigned females (*n* = 22; *M* = 0.44; *SD* = 0.28), indicating a stronger implicit gender self-concept compared to the rest of the groups. In contrast to predictions, a nonsignificant difference in the strength of the implicit gender self-concept was observed between autistic cisgender birth-assigned females and either non-autistic transgender or autistic transgender birth-assigned females. Lastly, we did not find a significant difference in the strength of the implicit gender self-concept between non-autistic transgender and autistic transgender birth-assigned females.

***Block 2.3: Explicit Gender Self-Concept***

*Hypothesis 1.* Among birth-assigned males and females, we expected non-autistic individuals (cisgender and transgender) to show a significantly stronger explicit gender self-concept than autistic people (cisgender and transgender). A nonsignificant difference in the strength of explicit gender self-concept was expected between non-autistic cisgender and non-autistic transgender people, as well as between autistic cisgender and autistic transgender individuals (non-autistic cisgender = non-autistic transgender < autistic cisgender = autistic transgender).

**Analysis.**

A 2 (birth-assigned sex: male/female) $\times$ 2 (diagnostic category: non-autistic/autistic) $\times$ 2 (gender identity: cisgender/transgender) ANOVA was conducted on participant scores from the explicit measure of gender self-concept (note: scores were transformed to positive values, so that the higher the score the stronger the explicit gender self-concept, regardless of whether it is male or female). As shown in Table S5, the 3-way interaction was nonsignificant. Nonetheless, to test our hypotheses a series of planned *t*-tests was conducted. Results of the analyses are presented in Table S5.

| **Table S5** | | | | | | | |
| --- | --- | --- | --- | --- | --- | --- | --- |
| *Explicit Measure of Gender Self-Concept Analysis of Variance Results and Planned t-Tests* | | | | | | | |
| Measure | Effect | *F* | *p* | 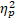 | Planned *t*-tests ^a^ | Cohen’s *d* | 95% CI |
| Explicit | Sex | 0.28 | .600 | .00 |  |  |  |
|  | Gender identity | 1.23 | .269 | .00 |  |  |  |
|  | Diagnostic category | 20.33 | <.001 | .06 |  |  |  |
|  | Sex $\times$ Gender identity | 4.51 | .034 | .01 |  |  |  |
|  | Sex $\times$ Diagnostic category | 9.48 | .002 | .03 |  |  |  |
|  | Gender identity $\times$ Diagnostic category | 35.10 | <.001 | .09 |  |  |  |
|  | Sex $\times$ Gender identity$\times$ Diagnostic category | 0.01 | .934 | .00 |  |  |  |
|  |  |  |  |  | **Birth-Assigned Males** |  |  |
|  |  |  |  |  | Non-autistic cis > Non-autistic trans *** | 1.01 | [0.56, 1.45] |
|  |  |  |  |  | Non-autistic cis > Autistic cis *** | 0.83 | [0.43, 1.23] |
|  |  |  |  |  | Non-autistic cis = Autistic trans | 0.39 | [-0.07, 0.84] |
|  |  |  |  |  | Non-autistic trans = Autistic cis | 0.04 | [-0.39, 0.46] |
|  |  |  |  |  | Non-autistic trans < Autistic trans * | -0.53 | [-1.02, -0.04] |
|  |  |  |  |  | Autistic cis < Autistic trans * | -0.47 | [-0.93, -0.01] |
|  |  |  |  |  | **Birth-Assigned Females** |  |  |
|  |  |  |  |  | Non-autistic cis > Non-autistic trans * | 0.36 | [-0.05, 0.78] |
|  |  |  |  |  | Non-autistic cis > Autistic cis *** | 1.41 | [0.99, 1.83] |
|  |  |  |  |  | Non-autistic cis > Autistic trans * | 0.57 | [0.10, 1.05] |
|  |  |  |  |  | Non-autistic trans > Autistic cis *** | 1.06 | [0.63, 1.49] |
|  |  |  |  |  | Non-autistic trans = Autistic trans | 0.21 | [-0.28, 0.69] |
|  |  |  |  |  | Autistic cis < Autistic trans *** | -0.84 | [-1.31, -0.36] |
| *Note.* Sex = birth-assigned sex; Cis = cisgender; Trans = transgender.  ^a^ Symbols for statistical significance denote results from one-tailed tests.  **p* < .05. ***p* < .01. ****p* < .001. | | | | | | | |

As expected, non-autistic cisgender birth-assigned males (*n* = 55; *M* = 4.84; *SD* = 0.74) scored significantly higher on the explicit task than non-autistic transgender (*n* = 37; *M* = 3.84, *SD* = 1.29) and autistic cisgender birth-assigned males (*n* = 50; *M* = 3.78; *SD* = 1.68). This indicates a stronger explicit gender self-concept among non-autistic cisgender birth-assigned males than among non-autistic transgender and autistic cisgender birth-assigned males. In contrast to predictions, a nonsignificant difference in the strength of the explicit gender self-concept was found between non-autistic cisgender and autistic transgender birth-assigned males (*n* = 29; *M* = 4.49; *SD* = 1.14). Also unexpectedly, non-autistic transgender birth-assigned males scored significantly lower on the task than autistic transgender birth-assigned males. Results suggest a weaker explicit gender self-concept among non-autistic transgender birth-assigned males than among autistic transgender birth-assigned males. A nonsignificant difference in the strength of the explicit gender self-concept was observed between non-autistic transgender birth-assigned males and autistic cisgender males. Furthermore, in contrast to predictions, non-autistic transgender birth-assigned males scored significantly lower on the task than non-autistic cisgender birth-assigned males, and autistic cisgender birth-assigned males scored significantly lower than autistic transgender birth-assigned males. Results indicate a weaker explicit gender self-concept among non-autistic transgender birth-assigned males than among non-autistic cisgender males and a weaker explicit gender self-concept among autistic cisgender males than among autistic transgender birth-assigned males.

As predicted, non-autistic cisgender birth-assigned females (*n* = 51; *M* = 4.89; *SD* = 0.94) scored significantly higher on the task than both autistic cisgender (*n* = 57; *M* = 2.98; *SD* = 1.64) and autistic transgender birth-assigned females (*n* = 27; *M* = 4.27; *SE* = 1.32). This denotes a stronger explicit gender self-concept among non-autistic cisgender birth-assigned females than among autistic cisgender and autistic transgender birth-assigned females. Also in keeping with predictions, non-autistic transgender birth-assigned females (*n* = 41; *M* = 4.52; *SD* = 1.13) scored significantly higher on the explicit task than autistic cisgender birth-assigned females, indicating a stronger explicit gender self-concept among non-autistic transgender birth-assigned females. Yet, a nonsignificant difference in the strength of the explicit gender self-concept was found between non-autistic transgender birth-assigned females and the autistic transgender birth-assigned females. In contrast to predictions, autistic transgender birth-assigned females scored significantly higher than autistic cisgender birth-assigned females, and non-autistic cisgender birth-assigned females scored higher than non-autistic transgender birth-assigned females. Results indicate a stronger explicit gender self-concept among autistic transgender birth-assigned females than among autistic cisgender birth-assigned females, and among non-autistic cisgender birth-assigned females than among non-autistic transgender birth-assigned females.

***Block 4: Association Analyses Between the Explicit and the Implicit Measure of Gender Self-Concept***

*Hypothesis 1*. Within each group, scores from the explicit measure of gender self-concept would correlate positively and significantly with scores from the implicit measure of gender self-concept.

*Hypothesis 2.* The above described correlations would be less strong among transgender individuals (non-autistic and autistic) than among cisgender people (non-autistic and autistic).

**Analysis.**

In keeping with the preregistration, a correlation analysis was conducted to examine the association between the explicit and the implicit measure of gender self-concept. As predicted, we found that, within each group, scores from the explicit measure were positively and significantly associated with scores from the IAT (non-autistic cisgender: *r*(97) = .79, *p* < .001 (one-tailed); autistic cisgender: *r*(89) = .72, *p* < .001 (one-tailed); non-autistic transgender: *r*(72) = .65, *p* < .001 (one-tailed); autistic transgender: *r*(46) = .63, *p* < .001 (one-tailed). Results suggest that participants’ explicit gender self-concept was in keeping with their implicit gender self-concept. That is, participants who identified explicitly with female groups identified implicitly with female groups as well, and participants who identified explicitly with male groups identified implicitly with male groups as well.

Next, a series of Fisher’s *Z* tests was conducted to examine between-group differences in the magnitude of the explicit $\times$ implicit relation. As expected, the association between the explicit and the implicit measure of gender self-concept was stronger among non-autistic cisgender people than among non-autistic (*z* = 1.81, *p* = .035, one-tailed) and autistic transgender individuals (*z* = 1.80, *p* = .036, one-tailed). Contrary to predictions, there was not a significant difference in the magnitude of the association between autistic cisgender and either non-autistic (*z* = 0.77, *p* = .220, one-tailed) or autistic transgender individuals (*z* = 0.90, *p* = .183, one-tailed).

***Block 5: Association Analyses Between Measures of Gender Self-Concept, and Current Gender Dysphoric Feelings and Recalled Gender-Typed Behavior***

*Hypothesis 1.* Within cisgender individuals (non-autistic and autistic), scores from the explicit and implicit measure of gender self-concept would be positively and significantly correlated with GIDYQ score and RCGI score (stronger explicit and implicit identification with gender groups = less current gender dysphoric feelings and more recalled gender-typed behavior).

*Hypothesis 2.* Within transgender individuals (non-autistic and autistic), scores from the explicit and implicit measures of gender self-concept would be negatively and significantly correlated with GIDYQ score and RCGI score (weaker explicit and implicit identification with gender groups of birth-assigned sex = more current gender dysphoric feelings and less recalled gender-typed behavior).

*Hypothesis 3.* The above described correlations would be less strong among transgender individuals (non-autistic and autistic) than among cisgender people (non-autistic and autistic).

**Analysis.**

In keeping with the preregistration, a series of correlation analyses was conducted examining the relations between the explicit and implicit measure of gender self-concept, and current gender dysphoric feelings (measured using the GIDYQ) and recalled gender-typed behavior (measured using the RCGI). Please note that scores from the explicit and implicit measure of gender self-concept were transformed to positive values, so that higher scores denote a stronger gender self-concept, regardless of whether it is male or female. As predicted, the strength of the explicit gender self-concept was positively and significantly related to GIDYQ score, *r*(103) = .37, *p* < .001 (one-tailed) and RCGI score, *r*(103) = .21, *p* = .017 (one-tailed) among non-autistic cisgender people. The strength of the explicit gender self-concept was also positively and significantly correlated to GIDYQ score, *r*(104)= .72, *p* < .001 (one-tailed) and RCGI score, *r*(104) = .56, *p* < .001 (one-tailed) among autistic cisgender people. Results suggest that the stronger the explicit identification of cisgender people (non-autistic and autistic) with the gender groups associated with their birth-assigned sex, the less their current gender dysphoric feelings tended to be and the more gender-typed behavior they recalled from childhood.

We also found that among non-autistic transgender people, the strength of the explicit gender self-concept was negatively and significantly related to GIDYQ score, *r*(76) = -.31, *p* = .003 (one-tailed) and RCGI score, *r*(76) = -.22, *p* = .026 (one-tailed). The correlation between the strength of the explicit gender self-concept and GIDYQ score was also negative and significant among autistic transgender people, *r*(54) = -.44, *p* < .001 (one-tailed). Contrary to predictions, the strength of the explicit gender self-concept was not significantly associated with RCGI score among autistic transgender people, *r*(54) = -.14, *p* = .161 (one-tailed). Results suggest that the weaker the explicit identification of transgender people (non-autistic and autistic) with the gender groups associated with their birth-assigned sex, the more their current gender dysphoric feelings tended to be, and the weaker the explicit identification of non-autistic transgender people with the gender groups associated with their birth-assigned sex, the less gender-typed behavior they recalled from childhood.

A series of Fisher’s *Z* tests was conducted to examine between-group differences in the magnitude of the associations. Contrary to predictions, neither the strength of the explicit gender self-concept × GIDYQ correlation nor the strength of the explicit gender self-concept × RCGI correlation differ significantly between non-autistic cisgender and non-autistic transgender people (*z* = 0.48, *p* = .314, one-tailed and *z* = -0.10, *p* = .459, one-tailed, respectively). Also contrary to predictions, neither the strength of the explicit gender self-concept × GIDYQ correlation nor the strength of the explicit gender self-concept × RCGI correlation differ significantly between non-autistic cisgender and autistic transgender people (*z* = -0.45, *p* = .325, one-tailed and *z* = 0.43, *p* = .333, one-tailed). As predicted, however, both the explicit gender self-concept × GIDYQ correlation and the explicit gender self-concept × RCGI correlation were significantly larger among autistic cisgender people than among non-autistic transgender (*z* = 3.89, *p* < .001, one-tailed and *z* = 2.66, *p* = .004, one-tailed, respectively) and autistic transgender people (*z* = 2.60, *p* = .005, one-tailed and *z* = 2.91, *p* = .002, one-tailed, respectively).

Furthermore, as predicted, there was a positive and significant association between the strength of the implicit gender self-concept and GIDYQ score, *r*(96) = .18, *p* = .042 (one-tailed) among non-autistic cisgender individuals. Results suggest that the stronger the implicit identification of non-autistic cisgender people with the gender groups associated with their birth-assigned sex, the less their current gender dysphoric feelings. In contrast to predictions, the relation between the strength of the implicit gender self-concept and RCGI score was nonsignificant, *r*(96) = -.01, *p* = .446 (one-tailed).

In keeping with predictions, the strength of the implicit gender self-concept was also positively and significantly correlated with GIDYQ score, *r*(88) = .24, *p* = .012 (one-tailed) and RCGI score, *r*(88) = .28, *p* = .004 (one-tailed), among autistic cisgender people. Nonetheless, a series of partial correlations revealed that the shared variance between the strength of the implicit gender self-concept, and GIDYQ and RCGI can be attributed to the strength of the explicit gender self-concept [*r*(87) = -.03, *p* = .760 and *r*(87) = .12, *p* = .266, respectively]. Contrary to predictions, neither among non-autistic transgender (*df* = 72) nor among autistic transgender (*df* = 46) individuals was there a significant association between the strength of the implicit gender self-concept, and GIDYQ score and RCGI score (all *p*s ≥ .275, one-tailed). Given that only one of our predictions was confirmed, between-group differences in the magnitude of the associations were not examined.

***Block 6: Association Analyses Between AQ, and Explicit and Implicit Measures of Gender Self-Concept for Each Birth-Assigned Sex Separately***

*Hypothesis 1.* Among non-autistic cisgender birth-assigned males, AQ score would be positively and significantly associated with scores from the explicit and implicit measure of gender self-concept (mores ASD-like traits = weaker explicit/implicit male self-concept).

*Hypothesis 2.* Among non-autistic cisgender birth-assigned females, AQ score would be negatively and significantly associated with scores from the explicit and implicit measure of gender self-concept (more ASD-like traits = weaker explicit/implicit female self-concept).

**Analysis.**

In keeping with the preregistration, a series of correlation analyses between AQ score, and scored from the explicit and implicit measure of gender self-concept was conducted for non-autistic cisgender birth-assigned males and non-autistic cisgender birth-assigned females, separately. As predicted, the correlation between AQ score and the score from the explicit measure of gender self-concept was positive and significant among birth-assigned males, *r*(53) = .41, *p* = .001 (one-tailed), and negative and significant among birth-assigned females, *r*(49) = -.25, *p* = .036 (one-tailed). Results indicate that the more ASD-like traits a non-autistic cisgender birth-assigned male self-reported, the weaker his explicit male self-concept, and the more ASD-like traits a non-autistic cisgender birth-assigned female self-reported, the weaker her explicit female self-concept. Contrary to predictions, AQ score did not correlate with the IAT *D* score either among non-autistic cisgender birth-assigned males, *r*(48) = -.17, *p* = .127 (one-tailed), or among non-autistic cisgender birth-assigned females, *r*(47) = -.08, *p* = .286 (one-tailed).

***Block 7: Links Between Autistic-Like Traits, Gender Dysphoric Feelings, and Mentalizing***

*Hypothesis 1.* Within each group, AQ score would be negatively and significantly correlated with GIDYQ score (more ASD-like traits = more current gender dysphoric feelings).

*Hypothesis 2.* Within each group, RMIE score would be negatively and significantly correlated with AQ score (better mentalizing = fewer ASD-like traits).

*Hypothesis 3.* Within each group, RMIE score would be positively and significantly correlated with GIDYQ score (better mentalizing = less current gender dysphoric feelings).

**Analysis.**

In keeping with the preregistration, we conducted a series of correlation analyses examining the relations between AQ, GIDYQ, and RMIE within each group. As predicted, AQ score was negatively and significantly correlated with GIDYQ score, *r*(103) = -.36, *p* < .001 (one-tailed) among non-autistic cisgender individuals. Results indicate that the more ASD-like traits a person self-reported, the more their current gender dysphoric feelings.

As predicted, AQ score was also negatively and significantly correlated with GIDYQ score, *r*(54) = -.43, *p* = .001 (one-tailed) among autistic transgender people. Results indicate that the more ASD-like traits an autistic transgender person self-reported, the more their current gender dysphoric feelings. In contrast to predictions, however, AQ score was positively and significantly correlated with GIDYQ score among autistic cisgender people, *r*(104) = .40, *p* < .001 (one-tailed), indicating that the fewer ASD-like traits an autistic cisgender person self-reported, the more current gender dysphoric feelings they reported. Also in contrast to predictions, the relation between AQ score and GIDYQ score among non-autistic transgender individuals was nonsignificant, *r*(76) = -.12, *p* = .149 (one-tailed).

Next, we examined the relation between RMIE score and GIDYQ score. As predicted, performance on RMIE task was found to be positively and significantly associated with GIDYQ score among autistic cisgender individuals, *r*(104) = .51, *p* < .001 (one-tailed), suggesting that the better their mentalizing ability, the less their current gender dysphoric feelings. Contrary to predictions, the relation between RMIE score and GIDYQ score was nonsignificant among non-autistic cisgender people, *r*(103) = .03, *p* = .377 (one-tailed), non-autistic transgender people, *r*(76) = .11, *p* = .175 (one-tailed) and autistic transgender people, *r*(54) = -.01, *p* = .468 (one-tailed).

Lastly, we investigated the relation between RMIE score and AQ score. Contrary to predictions, performance on RMIE task did not correlate significantly with AQ score either among non-autistic cisgender people, *r*(104) = .001, *p* = .495 (one-tailed) or among autistic transgender individuals, *r*(54) = .02, *p* = .453 (one-tailed). A negative and significant correlation between RMIE and AQ was found among non-autistic transgender people, *r*(76) = -.27, *p* = .008 (one-tailed). Contrary to predictions, we also found a positive and significant relation between RMIE score and AQ score among autistic cisgender individuals, *r*(105) = .27, *p* = .002 (one-tailed), suggesting that the better the mentalizing ability of autistic cisgender people, the fewer their ASD-like traits. Given that results were mostly out of keeping with predictions, no further analyses were conducted.

1. **Statistical Analyses Including Participants Whose Error Rate in the Critical Blocks of the IAT Exceeded 20%**

All the analyses included in the manuscript were reconducted including all participants, regardless of their error rate in the critical blocks of the IAT. Results of the analyses all presented below.

***Association Analysis Between AQ and Performance on the Implicit Measure of Gender Self-Concept***

When all participants included in the analysis, the relation between AQ and the strength of the implicit gender self-concept remained nonsignificant, *r*(104) = -.05, *p* = .299 (one-tailed), among non-autistic cisgender people.

***Performance on the Implicit Measure of Gender Self-Concept***

A 2 (birth-assigned sex: male/female) × 2 (diagnostic category: non-autistic/autistic) × 2 (gender identity: cisgender/transgender) ANOVA was conducted on participant scores from the IAT. Just as in the reduced sample, significant main effects were detected for birth-assigned sex, *F*(1, 338) = 4.34, *p* =.038, $\eta_{p}^{2}$ = .01, gender identity, *F*(1, 338) = 5.77, *p* =.017, $\eta_{p}^{2}$ = .02, and diagnostic category, *F*(1, 338) = 11.78, *p* =.001, $\eta_{p}^{2}$ = .03. The analysis also yielded a significant birth-assigned sex $\times$ gender identity interaction, *F*(1, 338) = 250.51, *p* < .001, $\eta_{p}^{2}$ = .43, a significant gender identity $\times$ diagnostic category interaction, *F*(1, 338) = 4.37 *p* = .037, $\eta_{p}^{2}$ = .013, and a nonsignificant birth-assigned sex $\times$ diagnostic category interaction, *F*(1, 338) = 0.20, *p* =.657, $\eta_{p}^{2}$ = .00. The *only* result that changed substantively in the full sample was the 3-way interaction. In the reduced sample, the 3-way interaction was nonsignificant, whereas in the full sample it was significant.

Breaking down the three-way interaction, a simple effects analysis of birth-assigned sex within gender identity and diagnostic category indicated that just as in the reduced sample the IAT was sensitive to gender identity differences. That is, among non-autistic and autistic cisgender individuals, birth-assigned females scored significantly higher on the IAT than birth-assigned males, whereas among non-autistic and autistic transgender individuals, birth-assigned females scored significantly lower than birth-assigned males (all *p*s < .001, all $\eta_{p}^{2}$s ≥ .07).

Next, we conducted a simple effects analysis of diagnostic category within birth-assigned sex and gender identity. Similar to the reduced sample, we found that autistic cisgender females achieved a significantly lower *D* score on the IAT than non-autistic cisgender females (*p* < .001, one-tailed, $\eta_{p}^{2}$ = .07) indicating a weaker implicit female self-concept. We also found that autistic and non-autistic transgender adults displayed an implicit gender self-concept that was in keeping with their experienced/reported gender, rather than birth-assigned gender, to the same degree (all *p*s ≥ .060, all $\eta_{p}^{2}$s ≤ .01). The only result that changed substantively in the full sample was the difference in *D* score between autistic and non-autistic cisgender birth-assigned males. In the reduced sample, autistic cisgender birth-assigned males scored significantly lower on the IAT than non-autistic cisgender birth-assigned males, whereas a nonsignificant between-group difference was found in the full sample (*p* = .078, one-tailed, $\eta_{p}^{2}$ = .01).

1. **Detailed Description of the RMIE and the Self-Report Measures Employed in This Study**

***Reading the Mind in the Eyes***

The Reading the Mind in the Eyes test (RMIE; Baron‐Cohen, Wheelwright, Hill, et al., 2001) is a reliable and widely used measure of adult mentalizing in clinical and nonclinical populations (e.g., Domes et al., 2007; Kelemen et al., 2005). Participants are presented with a series of 36 photographs each showing the eye region of people and they are asked to choose which of the four presented words best describes the emotional/mental state of the depicted person. Scores range from zero to 36, with higher scores indicating better mentalizing ability. In most studies, the test-retest reliability of the task is over .60 (e.g., Dehning et al., 2012; Fernández-Abascal et al., 2013; Voracek & Dressler, 2006). Specifically, Fernández-Abascal et al. (2013) reported an intraclass coefficient of .63 for 1 year test-retest reliability. Performance on the task clearly distinguishes autistic from non-autistic participants (e.g., Nicholson et al., 2019), and it is positively correlated with performance on other measures of mentalizing, even after the influence of IQ is controlled (e.g., Jones et al., 2018).

***Autism-Spectrum Quotient***

The Autism-Spectrum Quotient (AQ; Baron-Cohen, Wheelwright, Skinner, et al., 2001) is a reliable and widely used 50-item self-report measure of ASD-like traits. Participants are asked to indicate their level of agreement with a number of statements (e.g., “I find it difficult to imagine what it would be like to be someone else”) using 4-point Likert scale that ranges from “definitely agree” to “definitely disagree”. Scores range from zero to 50, with higher scores denoting more ASD-like traits. A score of ≥ 26 is the cut-off that denotes clinically significant levels of ASD-like traits. The sensitivity of the cut-off ranges from 88-95% in people with a diagnosis of ASD and the specificity ranges from 52-80% in people from the general population (Booth et al., 2013; Woodbury-Smith et al., 2005). AQ shows acceptable test-retest reliability with scores ranging from .70 to .95 (e.g., Baron-Cohen, Wheelwright, Skinner, et al., 2001; Broadbent et al., 2013) and shows convergent validity with the Social Responsiveness Scale in clinical (*r* = .64; Armstrong & Iarocci, 2013) and nonclinical samples (*r* = .55; Ingersoll et al., 2011).

***Gender Identity/Gender Dysphoria Questionnaire for Adolescents and Adults***

The Gender Identity/Gender Dysphoria Questionnaire for adolescents and adults (GIDYQ; Deogracias et al., 2007) is a 27-item reliable self-report measure that taps upon gender identity and gender dysphoria. Participants respond to a series of questions about their feelings, wishes, thoughts, and behaviors regarding their birth-assigned sex and their experienced/self-reported gender identity (e.g., “In the past 12 months, have you felt uncertain about your gender, that is, feeling somewhere in between a woman and a man?”) using a 5-point scale. Mean scores range from 1 to 5, with lower scores indicating more gender dysphoric feelings. A mean score of ≤ 3 is the cut-off that denotes clinically significant levels of gender dysphoria (Deogracias et al., 2007). The sensitivity of the cut-off ranges from 90-100% among people with a diagnosis of gender identity disorder and its specificity from 99.7-100% among a university students sample and cisgender adolescent and adults (Deogracias et al., 2007; Singh et al., 2010). GIDYQ also shows excellent internal consistency (Cronbach’s Alpha = .97; Deogracias et al., 2007). Participants completed the version of the questionnaire that was consistent with their birth-assigned sex.

***The Recalled Childhood Gender Identity/Gender Role Questionnaire***

The Recalled Childhood Gender Identity/Gender Role Questionnaire (RCGI; Zucker et al., 2006) is a 23-item self-report measure that provides a retrospective assessment of sex-typed behavior and closeness to parents (e.g., “As a child, my favorite playmates were: a. always boys, b. usually boys, c. boys and girls equally, d. usually girls, e. always girls, f. I did not play with other children”). In the current study, we used only the 18 items that assess gender role behavior and gender identity. Mean scores range from 1 to 5, with lower scores denoting less gender-typed behavior recalled from childhood. RCGI shows convergent validity with the GIDYQ, *r* = .70 (Singh et al., 2010). Participants completed the version of the questionnaire that was consistent with their birth-assigned sex.

**References**

Armstrong, K., & Iarocci, G. (2013). Brief report: the autism spectrum quotient has convergent validity with the social responsiveness scale in a high-functioning sample. *Journal of Autism and Developmental Disorders, 43,* 2228-2232.

Baron‐Cohen, S., Wheelwright, S., Hill, J., Raste, Y., & Plumb, I. (2001). The “Reading the Mind in the Eyes” test revised version: A study with normal adults, and adults with Asperger syndrome or high‐functioning autism. *Journal of Child Psychology and Psychiatry, 42*, 241-251.

Baron-Cohen, S., Wheelwright, S., Skinner, R., Martin, J., & Clubley, E. (2001). The autism-spectrum quotient (AQ): Evidence from asperger syndrome/high-functioning autism, males and females, scientists and mathematicians. *Journal of Autism and Developmental Disorders, 31*, 5-17.

Booth, T., Murray, A. L., McKenzie, K., Kuenssberg, R., O’Donnell, M., & Burnett, H. (2013). Brief report: An evaluation of the AQ-10 as a brief screening instrument for ASD in adults. *Journal of Autism and Developmental Disorders, 43*, 2997-3000.

Broadbent, J., Galic, I., & Stokes, M. A. (2013). Validation of autism spectrum quotient adult version in an Australian sample. *Autism Research and Treatment, 2013,* 1-7.

Dehning, S., Girma, E., Gasperi, S., Meyer, S., Tesfaye, M., & Siebeck, M. (2012). Comparative cross-sectional study of empathy among first year and final year medical students in Jimma University, Ethiopia: Steady state of the heart and opening of the eyes. *BMC Medical Education, 12,* 1-12. <https://doi.org/10.1186/1472-6920-12-34>

Deogracias, J. J., Johnson, L. L., Meyer-Bahlburg, H. F., Kessler, S. J., Schober, J. M., & Zucker, K. J. (2007). The gender identity/gender dysphoria questionnaire for adolescents and adults. *Journal of Sex Research, 44*, 370-379.

Domes, G., Heinrichs, M., Michel, A., Berger, C., & Herpertz, S. C. (2007). Oxytocin improves “mind-reading” in humans. *Biological Psychiatry, 61,* 731-733.

Fernández-Abascal, E. G., Cabello, R., Fernández-Berrocal, P., & Baron-Cohen, S. (2013). Test-retest reliability of the ‘Reading the Mind in the Eyes’ test: a one-year follow-up study. *Molecular Autism, 4,* 1-6. <https://doi.org/10.1186/2040-2392-4-33>

Greenwald, A. G., McGhee, D. E., & Schwartz, J. L. (1998). Measuring individual differences in implicit cognition: the implicit association test. *Journal of Personality and Social Psychology, 74*, 1464-1480.

[https://doi.org/10.1002/aur.1873](https://doi.org/10.1002/aur.1873%20)

Ingersoll, B., Hopwood, C. J., Wainer, A., & Donnellan, M. B. (2011). A comparison of three self-report measures of the broader autism phenotype in a non-clinical sample. *Journal of Autism and Developmental Disorders, 41,* 1646-1657.

Jones, C. R., Simonoff, E., Baird, G., Pickles, A., Marsden, A. J., Tregay, J., Happé, F., & Charman, T. (2018). The association between theory of mind, executive function, and the symptoms of autism spectrum disorder. *Autism Research, 11*, 95-109.

Kelemen, O., Erdélyi, R., Pataki, I., Benedek, G., Janka, Z., & Kéri, S. (2005). Theory of mind and motion perception in schizophrenia. *Neuropsychology, 19,* 494-500.

Mervis, C. B., & Klein-Tasman, B. P. (2004). Methodological issues in group-matching designs: α levels for control variable comparisons and measurement characteristics of control and target variables. *Journal of Autism and Developmental Disorders, 34*, 7-17.

Nicholson, T., Williams, D., Carpenter, K., & Kallitsounaki, A. (2019). Interoception is Impaired in Children, But Not Adults, with Autism Spectrum Disorder. *Journal of Autism and Developmental Disorders, 49,* 3625-3637.

Singh, D., Deogracias, J. J., Johnson, L. L., Bradley, S. J., Kibblewhite, S. J., Owen-Anderson, A., Peterson-Badali, M., Meyer-Bahlburg, H. FL., & Zucker, K. J. (2010). The gender identity/gender dysphoria questionnaire for adolescents and adults: Further validity evidence. *Journal of Sex Research, 47*, 49-58.

Voracek, M., & Dressler, S. G. (2006). Lack of correlation between digit ratio (2D: 4D) and Baron-Cohen’s “Reading the Mind in the Eyes” test, empathy, systemising, and autism-spectrum quotients in a general population sample. *Personality and Individual Differences, 41*, 1481–1491.

Woodbury-Smith, M. R., Robinson, J., Wheelwright, S., & Baron-Cohen, S. (2005). Screening adults for Asperger syndrome using the AQ: A preliminary study of its diagnostic validity in clinical practice. *Journal of Autism and Developmental Disorders, 35,* 331-335.

Zucker, K. J., Mitchell, J. N., Bradley, S. J., Tkachuk, J., Cantor, J. M., & Allin, S. M. (2006). The recalled childhood gender identity/gender role questionnaire: Psychometric properties. *Sex Roles, 54*, 469-483.
